# Discriminating activating, deactivating and resistance variants in protein kinases

**DOI:** 10.1186/s13073-025-01564-z

**Published:** 2025-10-28

**Authors:** Gurdeep Singh, Torsten Schmenger, Juan Carlos Gonzalez-Sanchez, Anastasiia Kutkina, Nina Bremec, Gaurav D. Diwan, Pablo Mozas, Cristina López, Reiner Siebert, Rocio Sotillo, Robert B. Russell

**Affiliations:** 1 BioQuant & Biochemistry Center, Heidelberg University, Im Neuenheimer Feld 267, Heidelberg, 69121 Germany; 2 Department of Hematology, Hospital Clínic de Barcelona, Barcelona, Spain; 3 Departament de Fonaments Clínics, Fundació de Recerca Clínic Barcelona-Institut d’Investigacions Biomèdiques August Pi I Sunyer (FRCB-IDIBAPS), Facultat de Medicina I Ciències de La Salut, Universitat de Barcelona, Barcelona, Spain; 4 Centro de Investigación Biomédica en Red de Cáncer (CIBERONC), Madrid, Spain; 5 Institute of Human Genetics, Ulm University and Ulm University Medical Center, Ulm, 89081 Germany; 6 Division of Molecular Thoracic Oncology, German Cancer Research Center (DKFZ), Translational Lung Research Center (TLRC), German Center for Lung Research (DZL), Heidelberg, 69120 Germany

**Keywords:** Protein kinases, Genetic variants, Gain-of-function, Loss-of-function, Resistance, Machine learning, Variant pathogenicity prediction, Cancer genomics, Precision medicine

## Abstract

**Background:**

Distinguishing whether genetic variants in protein kinases cause gain or loss of function is critical in clinical genetics. In particular, gain (and not loss)-of-function variants are often immediately amenable to treatment by inhibitors, making their identification a potential boon to personalised medicine. Most existing computational methods for variant pathogenicity prediction simply distinguish damaging from benign variants and provide no further functional insights. Here, we present a data-driven approach that differentiates activating**,** deactivating, and resistance variants.

**Methods:**

To train and evaluate our method, we curated a dataset of 2505 variants (375 activating, 1028 deactivating, 98 resistance and 1004 neutral) across 441 kinases from the literature and public databases. Each variant was represented as a vector of sequence, evolutionary and structural features, which we then used to train machine learning models to distinguish among the four types of variants. The resulting predictors achieved excellent performance (mean AUC = 0.941). We tested a selection of variants by over-expression in T-REx-293 cells followed by gene expression or biochemical tests.

**Results:**

Applying the predictors to uncharacterised variants, we observed a strong enrichment of activating mutations in cancer genomes, deactivating variants in hereditary disease, and few of either in variants from healthy individuals. We experimentally validated several predicted activating variants from cancer samples. For p.Ser97Asn in PIM1, phosphorylation events suggested increased activity. For p.Ala84Thr in MAP2K3, gene expression and mitochondrial staining showed a reduction in mitochondrial function, the opposite effect of MAP2K3 deletions. We provide an online application that enables users to analyse any kinase-domain variant, obtain prediction scores and explore known nearby variants in other kinases.

**Conclusions:**

Our predictors, together with the rapid experimental validations, demonstrates a feasible strategy for identifying activating variants in kinases in a time frame that would enable clinical decisions.

**Supplementary Information:**

The online version contains supplementary material available at 10.1186/s13073-025-01564-z.

## Background

The growth in high-throughput sequencing in biomedicine presents clinicians and biomedical researchers with new challenges [1, 2]. Among these is the need to identify which of the hundreds or thousands of genetic variants detected in a typical sequencing experiment are associated with the disease under study. Variants of unknown significance (VUS) are currently the clear majority of variants found in somatic tumour sequencing or clinical genetics [3, 4] and the increased availability and affordability of sequencing suggests that this situation will persist for several years.

Computational tools for characterising variants go back to the 1990s and have relied heavily on sequence conservation, sometimes combined with other structure or sequence features (e.g. [5–7]), to assess whether a particular variant will affect protein function. Variants linked to diseases are typically characterised as ‘damaging’ or ‘pathogenic’ with few additional insights into their specific functional consequences. Certain more recent tools offer additional insights from protein structure (e.g. [8, 9]) or protein interactions (e.g. [10]), but usually provide limited resolution beyond noting that a variant lies at or near a protein interface or functional region.

Decades of molecular biology research have produced an abundance of results that can help interpret genetic variants in more detail. This includes known [11] and predicted (e.g. [12]) protein structures, post-translational modifications (e.g. [13]), proteomics, interaction discovery (e.g. [14]), and extensive manual curation on protein function (e.g. [15]). If appropriately integrated, these data can reveal the roles of particular protein regions or positions and the specific consequences of perturbing them. Moreover, proteins fortunately often fall into large families, allowing insights learned from one member to inform understanding of the others.

Here we focus solely on protein kinases, one of the most important families in biomedicine, and present an approach that supplements popular variant interpretation tools by predicting whether a variant in the protein kinase domain is activating, resistance-causing, deactivating, or neutral. Note that this goes beyond the goals of pan-proteome predictors of ‘pathogenicity’ or protein ‘damaging’ effects for variants. Kinases are one of the only families where there appear to be certain trends in terms of activating or resistance variants and with sufficient data to derive more-specific predictors. GTPases (particularly G-proteins and Ras-like proteins) and G-protein coupled receptors (GPCRs) are also possibilities, though for GTPases there are a few well-established key positions that suggest gain-of-function [16] and for GPCRs constitutive activation is comparatively rare (e.g. [17]). In the future, as more gain- or loss-of-function variants are characterised for other families, proteome-wide predictors might become feasible.

Unlike other kinase function predictors [18], our method does not require specific structural information or knowledge of inhibitors. Instead, it exploits a wide range of prior curated data on kinase-altering variants and post-translational modifications, and requires only the variant as input. To demonstrate that this computational approach can guide clinically relevant research, we experimentally tested (via over-expression in T-REx-293 cells) a selection of variants in the kinases PIM1, MAP2K3 and CHEK2 confirming altered function with two showing increased activity, an observation that can immediately suggest treatments. Our analytical and experimental approach, together with the web application, will be of immediate use for identifying functional kinase variants with potential treatment or diagnostic implications.

## Methods

### Human kinase set and alignment

We obtained a set of human proteins containing kinase domains by extracting annotations from UniProt [15] and HMMer [19] searches of Pfam [20] hidden Markov model profiles (Pkinase and PK_Tyr_Ser-Thr) against human UniProt sequences (Additional file 2: Table S1). For proteins with multiple kinase domains, we divided the protein sequence into segments corresponding to the number of domains present, ensuring that each segment encompassed the sequence specific to its corresponding domain. This led to a total of 517 kinase domains from 477 proteins. We defined a functional kinase set of 484 domains (454 proteins) to build profiles for aligning sequences by excluding kinases or domains marked as catalytically inactive or pseudokinase in UniProt (Additional file 2: Table S1).

We aligned the kinase sequences using the hmmalign tool from HMMER [19] (version 3.1b2) against their respective Pfam hidden Markov model profiles (Pkinase and PK_Tyr_Ser-Thr) and trimmed the regions that were outside of the kinase domain. We added a maximum of 30 residues to the N- and C-termini for each kinase sequence, stopping before this if a domain was present. We merged the two alignments using MAFFT [21] (v7.520) (Additional file 2: Table S2) and used the hmmbuild tool (symfrac=0) to construct a profile hidden Markov model of the merged alignment (Additional file 2: Table S3). The alignment is available at activark.russelllab.org/alignment [22].

### Variant sets

**Fig. 1:**
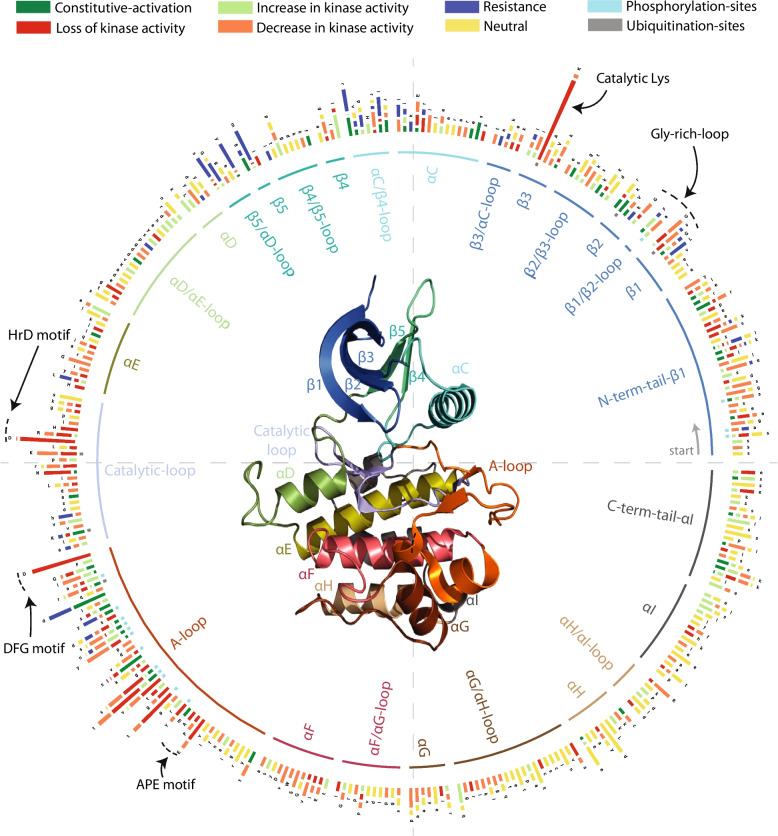
Distribution of known variants in human kinome. Plot showing the distribution of known functional and neutral variants in human kinases. Each bar corresponds to a position in the alignment constructed using human kinases. Variants are coloured based on their likely effect on the kinase activity: constitutively activated (dark green), increase (light green), loss (red), decrease (orange), and neutral (yellow). Resistance-causing variants are coloured blue. The total number of variants at an alignment position is log normalised (base 2) and divided based on the proportion of each variant type. Most conserved sites in the kinase domain are annotated with text and arrows. Phosphorylation and ubiquitination sites are highlighted in cyan and grey colours at the base of the bar. Structure in the centre refers to a canonical kinase domain and its secondary structure elements (INSR kinase, PDB ID: 1GAG)

We used a number of databases to obtain what we considered to be a reliable set of variants that modulate kinase function. Specifically, we identified variants that were activating, increasing, deactivating and decreasing kinase function; those that were associated with drug resistance; and a presumed neutral set (Additional file 1: Fig. S1; Additional file 2: Table S4). All variants were mapped to the alignments of the two kinase domains established above (Additional file 1: Fig. S2), though ultimately, we decided to use only the canonical kinase domain (Pkinase; Fig. 1).

#### Functional kinase variants and mutagenesis from UniProt

We downloaded all known mutagenesis or disease variant data (*n* = 6995) within the kinases above from the UniProt [15] database (version 2023_02), considering only single amino acid changes. We then went through the functional description (and often cited references) of each variant manually to identify clear evidence for kinase activity. We annotated a variant as ‘constitutively active’ or ‘increase’ if it led to constitutive activation or an increase in kinase activity, respectively. Similarly, we annotated a variant as ‘loss’ or ‘decrease’ if it led to complete loss or a decrease in kinase activity, respectively. This led to a set of 129 (96 in kinase domain) ‘constitutively active’, 203 (126) ‘increase’, 561 (435) ‘loss’ and 467 (283) ‘decrease’ variants in human kinases (Additional file 1: Fig. S1).

#### Additional activating variants from PubMed

We downloaded the entirety of PubMed [23] (up to 21 December 2022) and sought sentences in abstracts or titles containing a human gene name synonym (from UniProt) and matches to regular expressions for mutations (one or three-letter codes arranged before and after an integer). We then used BLAST [24] alignments of UniProt reviewed and un-reviewed sequences corresponding to human sequences sharing the same canonical gene name to identify putative sequences having the right wild-type amino acid at the right position and then to map these (where possible) back to the canonical UniProt reviewed entry. These variants were ranked according to the number of PubMed entries mentioning them and filtered as to whether they were kinases and whether the variant occurred within the kinase domain as identified by HMMer searches of the Pfam Pkinase hmm profiles.

These 84,802 putative variants were then cross-referenced with the PubMed entries matching searches for ‘constitutive* AND (activati* OR activate*)’ to give 607 candidate constitutively active variants. We then manually reviewed both lists to identify variants with clear evidence for each phenomenon and to remove spurious variants arising owing to chance (amino acid matches or spurious text matches). This identified 43 additional constitutively active variants, 25 of them within the kinase domain, bringing the total number of activating/increase variants to 375 (247 within the kinase domain) (Additional file 1: Fig. S1; Additional file 2: Table S4).

#### Candidate driver and resistance variants from COSMIC

We obtained all missense and confirmed somatic variants (and their sample counts) within the kinases above from the COSMIC [25] database (version 97, 29 Nov 2022), having first determined canonical UniProt sequence positions by aligning COSMIC to UniProt sequences with Muscle [26]. From the same COSMIC version, we also retrieved the set of 98 resistance variants (and the corresponding kinase inhibitor; 94 within the kinase domain), considering only confirmed somatic variants derived from tumour samples (Additional file 1: Fig. S1; Additional file 2: Table S4).

#### Neutral variants from gnomAD

We obtained naturally occurring variants within the kinases above from the gnomAD database [27] (version v2.1.1, 124,748 exomes) together with minor allele frequencies (MAF) and counts of heterozygous and homozygous instances. We mapped dbSNP variants in gnomAD to UniProt canonical kinase protein accessions. For instances with a MAF above 50%, variants were inverted and MAF recalculated (100%−MAF) so that they always refer to the less common allele. We defined neutral variants as those having a MAF > 0.001% and required all such variants to have at least two homozygous instances to avoid oddities related to exclusively heterozygous (and potentially disease) variants [28]. This led to a list of 1004 neutral variants with 203 within the kinase domain (Additional file 2: Table S4).

#### Training dataset

From the collections of kinase domain variants described above, we assembled a dataset for training the machine-learning models (Additional file 2: Table S5). A total of 375 activating/increase (A) and 1028 decrease/loss (D) variants came from the UniProt (347 A, 1028 D) and the PubMed (43 A) sets, 98 resistance (R) variants came from COSMIC and 1004 neutral (N) variants came from the gnomAD set. Restricting these to variants within our aligned kinase domain sequences resulted in a final training set of 1262 variants: 247 activating, 718 deactivating, 94 resistance and 203 neutral.

#### Test dataset

We also assembled an independent test set of kinase variants for the final evaluation of the machine learning predictors (Additional file 2: Table S6). Variants were collected from PubMed articles published after 21 December 2022 (after any dataset considered in the training set), using the same mining procedure described above, and any entries already present in the training data were excluded. Each variant was then manually annotated as activating, deactivating, or resistance based on the reported functional evidence. Of 527 kinase variants identified, 173 belonged to one or more of these three categories (62 activating, 56 deactivating and 58 resistance). Among these, 131 (76%) were located within the catalytic domain (41, 37 and 53, respectively), and 117 (35, 36, and 45) were within the set of 505 aligned kinases. We also attempted to update the data from UniProt, though we did not find a single additional annotated variant associated with increase, gain, decrease, loss or resistance that was not already in our training set or the PubMed testing set.

We collected neutral variants for the test set by updating our gnomAD set (20 April 2024) and excluding variants already present in the earlier dataset (as of 22 December 2022). This yielded 673 neutral variants, of which 166 (25%) were within the catalytic domain and 117 in the set of 505 aligned kinases. 

For additional tests, we generated five random variant sets by sampling 1000 variants randomly from all possible amino acid substitutions within kinase domain sequences that were not already classified as one of the other four categories.

### Post-translational modifications

We constructed a dataset of 7740 post-translational modifications (PTMs) in kinases with data from PhosphoSitePlus [29] (retrieved on 11 November 2022) and, to minimise false positives, excluding PTMs that were not supported by at least one low-throughput or two high-throughput citations (Additional file 2: Table S7). 

### Sequence conservation & structure features for machine learning

**Fig. 2:**
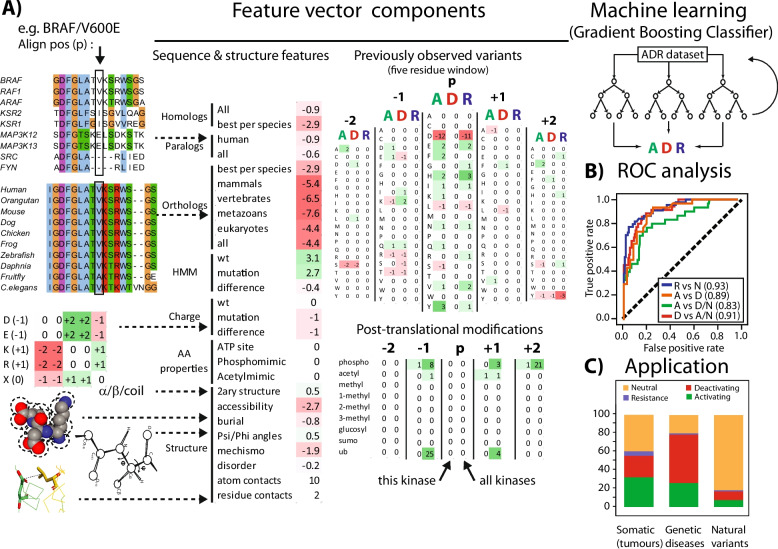
Machine learning workflow. **A** Workflow showing the procedure to construct a vector comprising the sequence conservation, structural and other information of a variant. For post-translational modifications and loss/gain of amino acids in known mutations, we also considered the information in the neighbouring residues (five residue window). **B** ROC curve showing performance of the predictors over a tenfold cross-validation during training. **C** Plot showing the fraction of variants predicted to be activating (green), deactivating (red), resistance (blue), and neutral (yellow) in three datasets: somatic variants (obtained from COSMIC; sample counts ≥ 5), variants associated to genetic diseases (obtained from UniProt) and natural variants (obtained gnomAD; AF ≥ 0.001; homozygous counts ≥ 2). Activating variants lead to constitutive activation or an increase in kinase activity. Deactivating variants leads to loss or a decrease in kinase activity

We used seven types of sequence, evolutionary and structural features to construct a vector for each variant within the kinase domain or within 30 residues of the N- or C-terminus in the datasets above (overview in Fig. 2A; full list in Additional file 3: Table S9).

#### One-hot encoding and charges of wild-type and mutated amino acids

We constructed two distinct vectors that represent the wild-type and mutated amino acids. Each amino acid was encoded by a binary vector of length 20, with a value of 1 at the corresponding position and 0 s elsewhere. We constructed an additional vector that encodes the charge on the wild-type and mutated amino acids.

#### Phosphomimetic or acetylation mimicking

A variant was considered phosphomimetic if the amino acid changed from a Ser (S) or Thr (T) to an Asp (D) or Glu (E), and acetylation mimicking if the amino acid changed from Lys (K) to Gln (Q).

#### ATP binding pocket

We calculated the number of known ATP binding sites at the position equivalent to the variant in the alignment. We obtained the list of known ATP binding sites in human kinases from UniProt (version 2023_02) (Additional file 2: Table S8).

#### Post-translational modification information

We incorporated known post-translational modification (PTM) information (Additional file 2: Table S7) of the variant position and its adjacent positions (window size = 5) as a feature vector, with a length equal to the number of possible PTM types (phosphorylation, acetylation, methylation, etc.). The presence of a specific PTM type was represented by 1, and otherwise by 0. We repeated the procedure to incorporate known PTM information at the alignment position equivalent to the variant position and its adjacent residues (window size = 5). Each element in the vector encoded the number of kinases harbouring the corresponding PTM type at the given position in the alignment.

#### Loss/gain of amino acids in known mutations

We incorporated the number of times an amino acid was observed to be a wild-type (loss) or mutated (gain) in a mutation type (i.e. activating, deactivating, and resistance) at the position equivalent to the variant (and its adjacent residues; window size = 5) in the alignment. We set the count initially to zero for all the amino acids at all alignment positions. For a loss of an amino acid at an alignment position in a mutation type, we decreased the corresponding count by 1 and increased it for a gain.

#### Conservation metrics across different sets of homologs

We extracted log scores for each amino acid and position from the profile hidden Markov model (see section above) and used the wild-type and mutated scores as features. We did the same for three additional alignments determined after pan-proteome comparisons and ortholog/paralog determination. We divided the orthologs based on the phylogeny into eukaryotes, metazoa, vertebrates, and mammals and used conservation across them as features. Specifically, this included conservation scores from three alignments (all homologs, best-per-species orthologs and exclusive paralogs used previously [28]).

#### Structural features

We used Alphafold2 [12] structures for each kinase to determine the secondary structure, accessibility and backbone psi/phi angles using DSSP. We used IUPred [30] to determine disorder scores. We scored intra-protein side-chain-to-side-chain contacts using Mechismo [8]. For all values, we determined log-odds values for each amino acid in each environment and used these and their mutant-wild-type differences as features (as described previously [28]). Note that we do not expect drastic changes in performance with other AlphaFold versions, as the majority of structures are made from high-identity templates (or even exact human structures). We opted to use AlphaFold only as it created a standard structural framework that integrated seamlessly with UniProt positions and gave homogenous structure data.

All datasets and features are available and can be downloaded as tar files [22].

### Machine learning predictors of kinase variant class

We created three predictors via machine learning (Scikit-learn library [31], Python version 3.10): a three-way Activating vs Deactivating vs Neutral predictor and two two-way predictors: Activating vs Deactivating and Resistance vs Neutral (Fig. 2). The rationale for this is discussed in the ‘Results’ section.

After exploring different methods (see below), we applied the Gradient Boosting Classifier, which involved building a predictive model by sequentially adding weak learners, where each new learner corrects errors made by its predecessors, and thus creating a combined model that involved constructing an ensemble of decision trees to predict the classification of variants according to all potentially applicable contrasts. We did this to test the ability of the system to distinguish variant sets from each other and to arrive at a final set of useful contrasts.

We used the ‘predict_proba’ method from the Scikit-learn library, which calculates the predicted class probabilities for a variant by averaging the probabilities across the trees in the forest. The class with the highest probability is considered the predicted class. To avoid bias arising from data imbalance, we used the ‘class_weight’ parameter in balanced mode.

We performed stratified tenfold cross-validation to tune the parameters of the predictors. The parameters tested included the number of boosting stages to perform {10, 25, 75, 100}, maximum depth of individual regression estimators {3,4,5}, minimum samples required at the leaf {3,5,7,10,12}, and the minimum number of samples required to split a node {3,5,7,10,12}. We determined the optimal parameters via grid search using the area under the receiver operating characteristic (AUC-ROC) and precision recall (AUPRC) curves as evaluation metrics. To ensure result robustness, we repeated the procedure 10 times for all the predictors and calculated the average AUC-ROC and standard deviation (Additional file 1: Fig. S3; Additional file 3: Table S10). We used standard performance metrics including the Matthews correlation coefficient (MCC), recall/sensitivity (REC/SEN) and specificity (SPE). To ensure the models did not overfit, we also performed a randomisation test [32] by repeating the above procedure with randomly shuffled labels (Additional file 1: Fig. S3; Additional file 3: Table S11).

We finally tested the performance of the predictors on 230 missense variants (Additional file 2: Table S6), missense mutations that were absent in the training set and are known to be both constitutively activating and resistant (Additional file 1: Fig. S4; Additional file 3: Table S12).

We selected the Gradient Boosting Classifier after testing a battery of machine learning methods using the same conditions described above (Random Forest, Gradient Boosting Classifier, Support Vector Machine, Neural Network, and Naive Bayes, and an ensemble of these methods). These approaches showed decreased performance during the cross-validation phase and, at best, similar performance on the test set (Additional file 1: Fig. S3E, S4; Additional file 3: Table S13). As Random Forest was only marginally worse than Gradient Boosting Classifier, we kept both sets of predictors in terms of data provided and results shown in the web application.

We calculated feature importance property using the Scikit-learn library (Additional file 1: Fig. S5).

Lastly, to enable biological interpretation (see the ‘Results’ section), we applied all our final models to predict the functional consequence of all (functionally characterised and uncharacterised) somatic variants in COSMIC and hereditary disease variants from UniProt, as well as all variants with no/low homozygous counts in gnomAD (Additional file 4: Table S16-S18).

### Evaluation of pathogenicity prediction

In order to evaluate our method in a more traditional variant pathogenicity prediction context (binary pathogenic/benign classification), we exclusively compared the performance of our ‘Activating, Deactivating or Resistance vs Neutral’ two-way model (a kind of forced pathogenicity predictor) to that of a large selection of methods, including state-of-the-art tools, on a test set. For this, we obtained the latest variant effect datasets available from dbnsfp.org, varity.varianteffect.org and tools.shenlab-genomics.org/tools/MAGPIE (29 August–1 September 2025). The test set consisted of 88 variants that were accessible by a single nucleotide change and for which all methods provided scores (Additional file 3: Table S14). We then calculated and compared all AUC scores (Additional file 1: Fig. S4; Additional file 3: Table S15). For methods with multiple predictors, we always selected the best AUC value. Note that for certain methods (e.g. EVE), datasets available were too limited to afford a reasonable comparison (i.e. they missed too many of the genes or variants in our test dataset).

### Web application

To complement the analysis, we developed a web application using the Flask web framework [33] and JavaScript libraries. The front end was implemented in HTML and CSS, and the back end in Python (v3.10). The web application is freely available at activark.russelllab.org, where users can explore prediction results with additional visualisation figures to help interpretation and all known functional information for the variant (from both training and test datasets). Moreover, they can download all underlying datasets for local analysis.

### Modulation of gene expression

We ordered gene sequences (Integrated DNA Technologies) containing the gene of interest with two stop codons flanked by the 5′ attB1 (5′-ACAAGTTTGTACAAAAAAGCAGGCTTC-3′) and 3′ attB2 sequences (5′-ACCCAGCTTTCTTGTACAAAGTGGT-3′). GatewayTM cloning into a pDest30 backbone (Thermo Fisher, #12301016) was performed following supplier instructions (Thermo Fisher, #11789020 and #11791020).

We then grew and maintained T-REx-293 cells (Thermo Fisher, #R71007) in T-REx Standard Culture Medium (high glucose DMEM, Thermo Fisher, #61965026) with 10% v/v FBS (Thermo Fisher, #26140079) and 100 U/mL penicillin and 100 µg/mL streptomycin (Thermo Fisher, #15140122). We transfected with pDest30 vectors to transiently overexpress wildtype or mutant genes in T-REx-293 cells, stably expressing the Tet repressor in the presence of 5 µg/mL blasticidin (Thermo Fisher, #R21001). Transfection was performed following the supplier’s instructions (Thermo Fisher, #L3000001) by transfecting 400,000 cells with 2.5 µg plasmid DNA.

After 96 h cells under selection pressure with 350 µg/mL geneticin (Thermo Fisher, #10131035), we induced expression using tetracycline (1 µg/mL, Thermo Fisher, #A39246) for 24 h and harvested for whole cell RNA isolation (Qiagen, #79254 and #74104).

### DNA damage induction assay

We transfected T-REx-293 cells with plasmids containing CHEK2 WT, p.Tyr86Ala or p.Lys373Glu to test the sensitivity to DNA damage [34]. After antibiotic selection, we seeded approximately 5 × 105 cells and induced them with tetracycline (1 µg/mL) for 24 h. We exposed cells to 400 µM H2O2 (Sigma-Aldrich, #1,086,001,000) [35] for 15 min and counted viable cells 24 h later (trypan blue, Thermo Fisher, #15,250,061) (Additional file 5: Tables S19-S20).

### Microarray analysis

We confirmed overexpression of target genes by q-RT-PCR (Thermo Fisher, StepOnePlus) and prepared 10 µL total RNA with a concentration of 50 ng/µL in biological replicates for microarray analysis (via the Genomics and Proteomics Core Facility, German Cancer Research Center, 69,120 Heidelberg, Germany). We deposited data in the Gene Expression Omnibus (GSE232293 [36]).

We analysed raw data using the R package maEndtoEnd [37] and assessed data quality via arrayQualityMetrics [38] removing any flagged chips before background correction and calibration. To remove low-intensity signals, we filtered data by setting a threshold based on median intensities. We defined contrast groups (mutation vs control, Additional file 1: Fig. S6–S10) and used empirical Bayes statistics to define differential expression (eBayes). For selected sets of significantly dysregulated genes (*P*adj-value ≤ 0.05) pathway, we performed enrichment analysis via gProfiler [39] (Additional file 1: Fig. SB). Detailed data are in Additional file 5: Table S21–23.

### Detection of kinase phosphosites

To test human MAPK14/p38 Thr180 and Tyr182 phosphorylation, we used an enzyme-linked immunosorbent assay (ELISA) kit (RayBiotech, #CBEL-P38-2). We grew 4×104 T-REx-293 cells and then transfected, selected and induced them (in triplicates) in wells of a 96-well plate (VRW, #734-0025), using a volume of 200 µL. The 96-well plate was coated with 20 µL of 0.1 mg/mL Poly-L-Lysine (Sigma-Aldrich, #P9155-5MG) for 2 h at RT prior to seeding. Twenty-four hours after tetracycline induction cells, we performed the ELISA procedure following the supplier’s protocol and measured absorbance at 450 nm (Tecan, Spark).

After removing the remaining solvent from the wells, we washed cells with deionised water and then stained them with 50 µL 0.1% (w/v) crystal violet (Sigma-Aldrich, #G2039-100G) for 20 min at RT. We then washed cells again with deionised water before de-staining with 100 µL 80% (w/v) ethanol for 30 min at RT. We measured absorbance at 590 nm to determine cell density.

We normalised the resulting α-phospho-p38 values based on the average signal and the relative cell density of each well determined by crystal violet staining. We determined outliers using a Z-score transformation:

$$Z=\frac{\overline{X }-\mu }{\sqrt{\frac{\sum_{i=1}^{n}{({x}_{i}-\mu )}^{2}}{n-1}}}$$

where *x* is the value of a single measurement and *µ* corresponds to the mean of an experimental group. We considered a *Z*-score ≥|3| to be an outlier and we removed the associated value. Raw and normalised results in Additional file 5: Tables S24–S25.

### Mitotracker staining

Cells were grown on coverslips (ibidi, #81158) and transfected as described above, followed by induction with tetracycline for 48 h. Culture medium was replaced with medium containing 100 mM MitoTracker Red CMXRos (Thermo Fisher, #M7512) for 15 min at 37 °C. Staining medium was then replaced with growth medium, and cells were imaged immediately on a Nikon Ti2 microscope (Ex 542/20, Em 620/52) (Additional file 1: Fig. S9; Additional file 5: Table S26).

An overview of all experiments performed and their results can be found in Additional file 5: Table S27.

## Results

### Overview of known functional variants

We extracted a set of 2505 missense variants from databases and the literature within 441 human kinases that we classified according to their impact on kinase enzymatic activity (Additional file 1: Fig. S1A; Additional file 2: Table S5). There were 375 variants in 140 kinases (247 or 66% in the kinase domain) missense variants that lead to constitutive activation or greatly increased activity (hereafter activating). This class of variant is most thought of in certain cancers, where a key (most often) somatic variant leads to greatly increased signalling as part of the pathogenesis of the disease and is thus often a drug target. However, our survey found more activating variants in genetic diseases (144 compared to 67 considering the 211 true disease variants; 126 of these are non-tumour/cancer predisposition variants; Additional file 2: Table S5), including kinases activating in Parkinson’s disease (kinase LRRK2), immunodeficiency (SYK), or Pfeiffer syndrome (FGFR2).

There were 1028 variants (718 or 70% in the kinase domain) in 289 kinases that have been shown to lead to either a complete loss of function or decreased enzyme activity (deactivating). This type of variant is less common in cancer, with just 27 of 233 (including seven tumour predisposition variants) of the true disease variants being from somatic tumours. Loss-of-function variants predominate in kinase variants causative of genetic diseases (Additional file 2: Table S5).

We considered 98 variants (94 or 96% in the kinase domain) in 17 kinases that arose owing to cancer drug resistance [25] (Additional file 1: Fig. S1A,D). These are more homogeneous than the other class, as resistance is highly context-dependent (i.e. on the inhibitor, the kinase mechanism, etc.) and they very often overlap (see below) with either activating or deactivating variants. We thus considered them separately from the other classes throughout the analysis.

Lastly, we defined a set of 1004 variants (203 or 20% in the kinase domain) in 304 kinases that we presumed to be neutral based on their constitutional appearance with a high frequency in healthy humans [27] (MAF ≥ 0.001; homozygous counts ≥ 2).

There were 168 instances of different variants of the same position in the same kinase having different known effects, of which most were related to phosphorylation (Additional file 2: Table S5). Most often this was to do with a mutation from serine/threonine to a negative charge (aspartate/glutamate), contrasting with the same position losing the hydroxyl (e.g. to an alanine). Essentially, whether a phosphosite was made constitutively on or lost altogether.

For evaluating our final machine learning models, we defined an additional testing set (Additional file 2: Table S6) derived from later PubMed entries (manually curated) and gnomAD data (see Methods). This consisted of 1193 variants (62 activating, 56 deactivating, 58 resistance and 673 neutral), of which 230 were inside the catalytic domain of our aligned set (see below) of kinases (35 activating, 36 deactivating, 45 resistance and 117 neutral; 3 activating and resistance overlapped). The above trends on the training set largely hold for the variants within this set.

We constructed an alignment of the catalytic domain plus maximally 30 residues at the N- and C-terminus for 464 human kinases (Methods) that we used to position all these variants onto a common positional frame. We also marked regions on this alignment according to the canonical functional regions of protein kinases [40] (Fig. 1) that we refer to below.

Inspection of the location of the different types of variants reveals immediate trends (Fig. 1). First, as mentioned above, functional variants are greatly enriched in the catalytic domain (Additional file 2: Table S4). This is true for activating, deactivating and resistance variants in both training and testing datasets, in addition to genetic disease variants from UniProt and somatic variants from COSMIC, both of which increase as evidence for disease/function increases (either evidence from UniProt or variant frequency in COSMIC). The opposite is true for neutral variants, which show a slight tendency to avoid the catalytic domain that increases slightly with the frequency of the variant in the population as might be expected if they were truly neutral (Additional file 2: Table S4). There is a small but interesting subset of functional variants outside of the catalytic domain, including some variants with very high sample counts in COSMIC. Most of these are Cysteine losses or gains in the extracellular part of transmembrane tyrosine kinases that have been shown to lead to activation by inter-subunit disulphide bond formation and thus constitutive dimerisation (e.g [41].).

Resistance and activating variants tend to avoid the most conserved and functionally core parts of the kinase catalytic domain (Additional file 1: Fig. S1B) and often overlap with each other. In contrast, deactivating variants very often hit key parts of the enzymatic machinery, particularly the catalytic Lysine and the Aspartate residues in the ‘HRD’ and ‘DFG’ motifs (Additional file 1: Fig. S1E). For instance, BTK p.Lys430Glu (catalytic Lys) abolishes kinase activity, leading to X-linked agammaglobulinemia, a rare genetic disorder characterised by the body’s inability to produce normal B cells [42], and DAPK3 p.Asp161Asn (in the DFG motif) greatly reduces kinase activity, promoting cell survival and cell proliferation in ovarian mucinous carcinoma [43]. We found 15 positions within the alignment where known activating and deactivating variants overlap (at least 2 counts of each type and at least 5 variants). 10 of these positions lie within the A-loop, and 2 each in the N- or C-terminal tails of the kinase domain. For example, one alignment position in the activation loop has 23 activating and 60 deactivating variants (Fig. 1). Inspection shows that these are almost always at phosphorylation sites, where most often activation is accomplished by mutation to a negative charge (Asp/Glu) and deactivation by a loss of the phosphorylatable residue (e.g. to Ala or similar) (Additional file 1: Fig. S1E). For example, in IKBKB p.Ser181Glu, mutating the uncharged Serine to a negative charge leads to full activation of its kinase activity and activation of the NF-kappa-B pathway [44], while LATS2 p.Ser872Ala (at the equivalent alignment position), mutating the phosphorylatable Serine to an unphosphorylatable Alanine, is reported to lead to loss of its tumour suppressor activity in mice [45].

There are also many variants outside of, but near the catalytic domain, particularly at the N- (76 variants) and C-terminal (61 variants) tails, with roughly the same proportion (of activating, deactivating, etc.) as the entire dataset, though with no resistance variants in the C-terminal tail (Fig. 1, Additional file 1: Fig. S1A, 1C). Just under half (43%) of these are at phosphosites, as might be expected, given that many kinases are phosphorylated in their tails as part of the activation process. Resistance mutations occur almost exclusively at or near the ATP binding pocket (N-lobe, catalytic and activation loops), and indeed, we found none in or after the C-lobe (Additional file 1: Fig. S1A, 1C).

We initially explored separate analyses of Tyrosine and Serine/Threonine kinases. However, we observed considerable overlap in the datasets in the two classes, for instance, in terms of where the variants were on the canonical kinase structure (Additional file 1: Fig. S2). Moreover, since certain datasets tend to skew more heavily to one class (e.g. resistance variants to Tyrosine kinases), we reasoned that the separation would also make our predictors (below) less effective owing to data paucity.

### Machine learning trained predictors for kinase variant function

The patterns described above suggested that a machine learning predictor of kinase variant type would be possible. Accordingly, we applied multiple machine learning algorithms (Gradient Boosting Classifier, Random Forest, Gaussian Naive Bayes, Support Vector Classifier, Multi-Layer Perceptron Classifier and an ensemble of these) to develop three contrasting predictors based on seven types of sequence and structural features (Fig. 2; Additional file 3: Table S9). Consistent with our observations, we only considered variants that are within the kinase domain or within 30 amino acids of the N- or C-terminus (excluding these residues that overlap with other domains; see the ‘Methods’ section).

We developed three predictors (one three-way and two binary) to address practical scenarios in kinase variant analysis. All models were trained on sequence- and structure-derived features from the activating, deactivating, resistance and neutral variants above (within the catalytic domain region) and then evaluated on a smaller, non-overlapping test set (Methods; Additional file 3: Table S10).

The activating, deactivating, or neutral (three-way) predictor is reflective of a situation where one does not know if a variant is functional at all, and thus, neutral variants must be distinguished. This could arise when sequencing identifies a novel variant (say a disease cohort), and one is not sure of its relevance (i.e. it could be neutral). This situation might also arise when looking for functional mutations (e.g. via site-directed mutagenesis) to design kinases to be activating or deactivating. The activating vs. deactivating (binary) predictor applies when one has a variant believed already to be functional (e.g. observed many times in a cohort or dataset, or predicted as ‘pathogenic’ or ‘damaging’ by existing predictors), but the specific effect on kinase function is unknown. In other words, we know the variant is important, but what is it doing to the enzyme? Lastly, the resistance vs neutral (binary) predictor exclusively identifies whether or not a variant confers resistance (Additional file 3: Table S10). We avoided contrasting resistance to activating or deactivating due to the considerable overlap between activating and resistance variants (above) and because resistance variants were from an entirely distinct source (Additional file 1: Fig. S2). Note that this also means that predictions of resistance should be considered alongside the other two predictors since there will necessarily be a tendency to predict many activating or deactivating sites as resistant. All three predictors showed strong performance when tested against random or neutral variants (Additional file 1: Fig. S4).

The best performance was achieved using the Gradient Boosting Classifier (GBC) with random forest (RF) being only marginally worse (Additional file 3: Table S13, Additional file 1: Fig. S3E) when considering ROC or Precision-Recall curves or associated confusion matrices. Unless otherwise noted, results in the following sections are reported from the best-performing GBC model. AUC values from ROC analysis during cross-validation (0.91–0.95) were comparable with those obtained on 145 variants from an independent test set absent from the training phase (0.80–0.93; Additional file 3: Table S10). The biggest difference seen when predicting activating vs neutral (0.91 training, 0.80 testing), which is possibly to do with the greater proportion of somatic variants in the training (155/502 = 30.9%) compared to the test set (73/297 or 24.6%).

As our dataset is unbalanced in terms of the number of each type of kinase variant (e.g. there are more than twice as many deactivating as activating variants), we also computed balanced accuracy (values between 0.72 and 0.85 for all predictors) and MCC (values between 0.38 and 0.69), which suggested good overall performance (Additional file 3: Table S10).

We also tested whether model performance was inflated by overlap between the training and test sets, specifically variants affecting the same residue but with different substitutions. The resulting differences were marginal (Additional file 1: Fig S4F). The difference between GBC and RF on the test set was also small, with the former better for most, but not all predictors (Additional file 1: Fig S4E, 4G).

When comparing against random variants (instead of neutrals), the performance is generally slightly worse (Additional file 1: Fig. S4A–4C). Notably, we saw good performance at predicting neutrals from random, which probably argues that random variants are a lot more likely to be functional (mostly deactivating). We did not pursue this further as we did not envision this to be a realistic scenario where random variants would be encountered (e.g. a research or clinical setting). Note that predictors of just ‘pathogenicity’ (e.g. AlphaMissense [6]) also perform worse when tested against random variants (see below).

The better performance for these decision tree-based approaches agreed with our impressions from the dataset analysis above; namely that there are diverse contextual reasons for a position to be activating or deactivating (see discussion of phosphosites above). This suggests that more additive approaches (e.g. Naive Bayes) would be less optimal to distinguish these sites.

The fact that predictions of resistance have such a strong performance is somewhat surprising as inhibitors vary greatly and variants can be highly specific to one of a set of similar drugs. However, there are nevertheless features of resistance variants that seem to distinguish them from others. It is clear that the data (from COSMIC) are biased towards ATP-site inhibitors, and it is likely that performance on non-canonical inhibitors would be worse.

We also compared our results to existing predictors of variant impact [5–7]. It is important to emphasise that these other predictors do not attempt to distinguish different types of functional variants (activating/deactivating/resistance), but rather to distinguish broadly structurally/functionally disruptive variants (i.e. including all three types of variants in one) from neutral. When grouping activating, deactivating and resistance together, mimicking the more general prediction of ‘pathogenicity’ used by other tools, our approach performs similarly to a selection of other predictors that easily provided non-SNV based protein changes on variants within the kinase domain, though clearly AlphaMissense is best overall (Additional file 1: Fig. S4H). Interestingly, PolyPhen2 and PMUT fared worse on activating and resistance variants when considered separately, probably as these are likely under-represented in sets of ‘damaging’ or ‘pathogenic variants used to train them. Note also that all methods did worse when considering random variants as opposed to neutrals (Additional file 1: Fig. S4A–4C). We also compared the approach to a wider set of 40 pathogenicity predictors by restricting the test dataset to the 88 variants that were accessible by a single nucleotide change (Additional file 3: Table S14–S15) with similar results. Our method performs as well (AUC 0.913) as the best predictors (ranked 12th of 41; mean of all methods AUC = 0.857), with the caveat that we were not able to assess whether the test dataset variants were in the training sets of the other methods. For some methods (e.g. MAGPIE [46]), the use of data from (e.g. OMIM) as features likely means that several of our functional test set variants were involved in training. It is crucial to emphasise that our goal is not to predict pathogenicity better than these predictors, but to distinguish more specific functional consequences that these generic predictors do not, and that the best-use case is to use our predictor alongside a more generic predictor of pathogenicity for variants that lie in the protein kinase domain (as indeed is often how clinically relevant candidate kinase variants are currently identified). Our own analysis (Additional file 1: Fig. S4H) suggests that AlphaMissense might give better results when attempting to discriminate activating from deactivating and neutral. Nevertheless, the fact that these results are similar to those from well-established methods gives us confidence in our machine learning strategy for our main three predictors. We also attempted to compare our predictions to an earlier method to predict kinase activating variants [18]; however, the need to specify particular experimental structures for each prediction made this problematic.

To validate that the predictors did not overfit, we conducted a randomisation test (see the ‘Methods’ section). The performance (Additional file 3: Table S11) of randomised predictors (AUC close to 0.5) was worse than the original predictors, suggesting that our approach is not susceptible to overfitting.

We used the Gini importance metric to evaluate feature importance (Additional file 1: Fig. S5). We found that while specific amino acid substitutions (one-hot encoding) did not overall heavily drive the predictions, certain changes involving distinct amino acid features like charges proved useful. The most important features for machine learning driving the predictions were found to be conservation across human kinases, homologs, paralogs, orthologs, and post-translational modification (PTM) information at the variant site. Interestingly, certain features have distinct contextual importance. Conservation across different sets of homologs (e.g. orthologs and paralogs) and PTMs is key to distinguishing activating and deactivating from neutral variants (Additional file 1: Fig. S5A), but insufficient for differentiating between activating and deactivating variants (e.g. consider variants occurring at phosphosites) (Additional file 1: Fig. S5B). For this, conservation across all kinases is highly relevant, since the most conserved positions are known to disrupt the kinase activity (e.g. catalytic Lys and DFG-motif) and are more likely to indicate deactivating positions, whereas positions conserved, for example, in mammalian orthologs (Additional file 1: Fig. S5B), could be either activating, resistance, or deactivating. We suspect that this is the reason why different conservation values play such distinct roles across the various predictors. We found the ATP binding information to be most relevant for resistance variants (Additional file 1: Fig. S5C), which is expected since most kinase inhibitors are known to be ATP-competitive [47].

It is informative to consider where the methods failed on the testing dataset. Considering the 36 activating variants, 3 were complete failures in the sense that activation was never the best prediction (A vs D vs N or A vs D). TNNI3K p.His592Tyr lies just C-terminal of the HRD (active site) motif, in a position that has only deactivating variants in other kinases, all of which are also a loss of a positive charge (Arg). It is clear that the loss of the partial charge in this variant is scoring well as a deactivation owing to the similarity between His and Arg. PINK1 p.Gly411Ala is similarly in a region of only deactivating variants in the training set just in the region between the A-loop and the APE-motif. Several of the previously known deactivating variants convert a polar to an Ala, and are indeed mostly site-directed mutagenesis results, which might be biasing the predictions. Lastly, the MAP2K1 p.Asn122Asp activating variant is predicted deactivating likely because of the deactivating variants NEK7 p.Asn90Lys/Arg/Ala suggesting a loss of Asn at this position to be detrimental.

The six deactivating variants (of 36) in the test set predicted to be activating show similar trends. For instance, CHEK2 the deactivating variant p.Glu321Ala (on the N-terminus of aE) is predicted as activating likely because of very similar variants known to be activating at the same alignment position, such as CAMK2A p.Glu109Asp and CAMK2B p.Glu110Lys, plus the fact that no deactivating variants are at or near this position in the training dataset. Similarly, the deactivating variant AKT1 p.Arg273Gln is predicted activating as all other losses of Arginine at this alignment position are activating or resistant, such as the paralog AKT2 p.Arg274His. The other four are at positions where very little is known regarding activation or deactivation.

The eleven resistance variants (of 55) predicted neutral were all within regions of the alignment for which little or most often no resistance variants had been seen in any other kinases. For several, there were also both activating and deactivating variants muddying the predictions. This highlights the general overlap of resistance positions and indeed the same variants with both activating and deactivating, and indeed the bias of resistance variants to the COSMIC database in the training set (see above). For the 27 (of 118) neutrals that were not predicted neutral, 13 were predicted activating (two also resistance) and 14 deactivating (one also resistance). Here it is more challenging to see any pattern, but it is interesting to note that some of these predictions are very strongly predicted as activating (8) or deactivating (2). These include predicted activating variants NEK1 p.Arg261His and BLK p.Arg359Cys that both have conflicting reports of pathogenicity (in gnomAD [27]), which raises the question as to whether they are truly neutral.

Overall, it is clear that the system is to some extent limited to the data used to train it. Positions in the kinase alignment that have limited information are harder to predict. This suggests that the method will improve as more data arrive to train the predictors. This moreover suggests that predictions should be considered in context. It is helpful to inspect other kinases to see whether the same or similar variants have been seen previously to have the predicted effect. To aid in these efforts and generally to provide visualisation and accessibility of the prediction results and known information about a given variant or mutation, we developed a web application (activark.russelllab.org), which allows users to input multiple variants, rank them according to different predictors and peruse them in depth alongside data from our curated variant set. For this purpose, we also developed a technique to rapidly annotate and display alignment sections around the variant of interest.

### Known and potentially new functional variants within existing datasets

We applied our approach to kinase variants from three large datasets: somatic cancer variants from COSMIC [25], hereditary disease variants from UniProt [15] and naturally occurring (healthy) variants from gnomAD [27]. Overall, the disease variant sets show enrichment in the kinase domain that increases as one raises the level of certainty (i.e., requiring an increasing number of COSMIC samples or evidence in UniProt annotations), while in contrast, neutral variants show a slight tendency to avoid the kinase domain (Additional file 2: Table S4). These observations support the general notion of a predictor to discriminate functional variants lying in the kinase domain.

The overall proportions of activating, deactivating and neutral predictions for these sets agreed with our expectations (Fig. 2C). The fraction of activating variants was more pronounced in the somatic variant dataset, in contrast to deactivating variants being significantly more predominant in hereditary diseases, reflecting the high proportion of kinases among oncogenes and the general tendency for hereditary diseases to lead to loss of function rather than gain. Reassuringly, natural variants had the greatest proportion of neutral predictions and very few activating or deactivating instances. Resistance variants (that were not already predicted as activating or deactivating) are nearly absent in hereditary diseases, with the greatest proportion in somatic and a smattering in hereditary variants.

Within somatic variants in COSMIC [25], deactivating variants from our curated set are comparatively rare, with only 20 observed in total (including 6 known tumour suppressors) across 187 samples, with very low individual sample counts (all variants have fewer than 50; 14 have fewer than 10; Additional file 4: Table S16). Surprisingly, known activating variants (even known somatic variants) also often have low sample counts in COSMIC. Although there are several that are seen thousands of times, mostly due to a predominance of tumours driven by well-known cancer driver genes (e.g. JAK2 p.Val617Phe, EGFR p.Leu858Arg, KIT p.Asp816Val, BRAF p.Val600Glu/Lys), nearly half (54/110) of both activating and resistance variants are seen with counts of 50 or fewer; just under a third (33/110) are seen with 10 counts or fewer (Additional file 4: Table S16). These include activating variants BRAF p.Leu597Val (seen 16 times) and MAP2K1 p.Gln56Pro (33 times), both of which are well-established oncogenic variants [48, 49]. This suggested the possibility that many other rare constitutively active variants might lie within the existing data with comparatively low sample counts. We explore some of these in greater detail below.

**Fig. 3:**
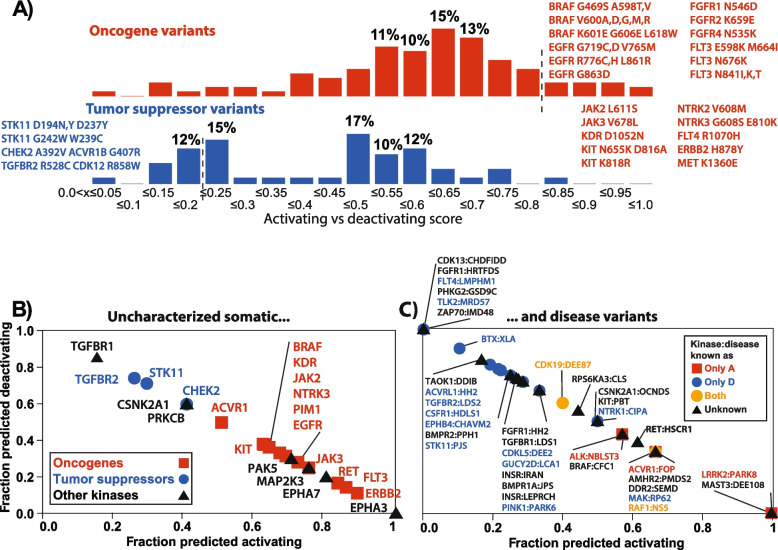
Application to uncharacterised variants somatic and genetic disease variants. **A** Histograms (top) showing distribution of Activating vs Deactivating scores for all variants reported in COSMIC for unambiguous oncogenes or tumour suppressors (COSMIC gene census) having at least 5 reported samples and excluding those already known to be activating/deactivating/resistance (346 variants from 38 oncogenes, 41 from 8 tumour suppressors). Predicted novel activating/deactivating variants (score ≥ 0.8 or ≤ 0.2) are also given. **B** Plot showing the fraction of deactivating/activating variants in those tumour suppressors (blue) and oncogenes (red) with at least 5 variants with at least 5 counts in COSMIC. Here variants are characterised as activating either if the A/D/N prediction shows ≥ 0.333 A or if the AIvDL prediction is higher than 0.7. The fraction reported is the fraction of the total predictions being activating/deactivating. **C** as for **B** but for kinase:disease pairs (gene name:disease abbreviation from UniProt/OMIM) extracted from genetic disease data within UniProt. Classified pairs are only shown if there were two or more known activating/deactivating variants to classify them as only-activating, only-deactivating or both. These and those lacking any prior known variants were plotted only if there were at least three predictions made (method as for **B**) of activating or deactivating among the unclassified variants

COSMIC also provided some additional insights into the nature of variants in kinases and some support for the efficacy of our predictor. Considering the set of kinases (of 68 in total) that are unambiguously assigned as oncogenes (45) or tumour suppressors (9) in the COSMIC Cancer Gene Census, and excluding variants already known to be deactivating or activating, we found a reasonable separation in activating or deactivating predictions when comparing oncogenes to tumour suppressors (Fig. 3A; note only three tumour suppressors and 12 oncogenes had at least four variants remaining after filtering knowns). Moreover, when considering the fraction of unclassified variants predicted as one or the other, it is clear that oncogenes have a higher fraction of activating and tumour suppressors of deactivating, as expected (Fig. 3B). This is interesting as information about the kinase itself was not part of the predictor and suggests that frequently observed variants in previously known oncogenes or tumour suppressors are likely to have the expected (i.e. activating or deactivating, respectively) effect.

This analysis also highlighted several very strongly predicting activating variants in well-established oncogenes or the opposite in tumour suppressors (red and blue text in Fig. 3A). This has clear implications for both types of cancer genes. Naturally, activating variants can lead to personalised treatments in the form of specific kinase inhibitors. However, knowledge of deactivating variants in tumour suppressors can also have important implications for diagnostics; for instance, both CHEK2 and STK11 variants are difficult to assess in the context of hereditary cancers (e.g. [50]). We also observed a handful of instances where an oncogene has clearly deactivating, frequently observed variants, notably BRAF p.Asp594Gly/Gln in the DFG motif and p.Lys483Glu at the catalytic lysine, though these are well understood (and genuine exceptions) [51].

Considering kinases not in the Cancer Gene census and for which sufficient variants are seen in COSMIC (black triangles in Fig. 3B), the predicted fraction of activating/deactivating variants argues that CSNK2A1, PRKCB and TGFBR1 are more likely to be tumour suppressors and PAK5, MAP2K3, EPHA3 and EPHA7 oncogenes. This largely agrees with what has been recently postulated in the literature except for EPHA7, which is most often referred to as a tumour suppressor, though it also has roles in promoting tumours [52]. It is important to emphasise that these findings are biased towards the data that are currently in the COSMIC database.

We also performed a similar analysis of all kinase hereditary disease variants in the UniProt database that are not already annotated as activating or deactivating (Fig. 3C; Additional file 4: Table S17). We first identified kinase:disease pairs with at least two variants previously characterised as activating or deactivating and for which at least three predictions were made by the system. This gave three pairs being only activating, 13 only deactivating and two having both. When considering predictions for the uncharacterised variants (excluding those variants known to be activating/deactivating), we see a reasonable separation between only-activating and deactivating (Fig. 3C). As above for cancer genes, a number of kinase:disease pairs lacking prior characterisation fall into discrete regions of the plot, thus suggesting whether the pathology is dictated by kinase activation or deactivation. For example, mutations in Microtubule-associated serine/threonine-protein kinase 3 (MAST3) are implicated in developmental and epileptic encephalopathy (DEE108) [53]. We predicted 6 out of 6 variants in MAST3 linked to DEE108 as activating (albeit with one marginal activating/neutral prediction). Two of these (p.Gly510Ser and p.Gly515Ser) are associated with increased phosphorylation of a MAST3 target protein and thus are likely a gain of function [53]. In contrast, we predict 5 out of 5 variants in FGFR1 associated with Hartsfield syndrome (HRTFDS) to be deactivating, and inspection shows four very likely are, as they involve highly conserved active site residues such as glycines in the N-terminal Glycine-rich loop (p.Gly490Arg) or within the catalytic loop/HRD motif (p.Asp623Tyr, p.Arg627Thr, p.Asn628Lys).

Among variants seen in healthy humans (gnomAD [27]) we found 53,690 kinase domain variants of which 194 display a MAF ≥ 1%. From those, we found 7 variants with ≤ 5 homozygous counts (Additional file 4: Table S18). The high allele frequency together with depleted homozygous counts argues that such variants may be functional [28]. For example, p.Val124Gly in CDK1 is conserved across orthologs, lies within the activation loop and is predicted to be deactivating, as it is only 2 positions N-terminal of the HRD motif, where many deactivating mutations are seen in other kinases (STK11 [54], CSFR1 [55] and others). Elsewhere, the gain of a negative charge in the TNK1 variant p.Ala299Asp is predicted to be activating as it is adjacent to known phosphosites in AKT1 and IKBKB in the kinase family sequence alignment (Additional file 2: Table S7). Our predictions together with the absence/low frequency of homozygous counts suggest that these and other variants could be functional.

### Experimental tests of selected kinase variant predictions

To illustrate how the predictor can be used to determine interesting candidates for further experimental study, we selected 9 variants from four kinases for rapid experimental tests (overview in Additional file 5: Table S27).

Seven of these came from the COSMIC analysis above, including four predicted activating variants (one of which was already known) and three predicted deactivating variants. We added two additional deactivating variants in the form of well-known alanine variants of key active site residues. We could not identify any clear data source of previously unknown resistance variant candidates on which to perform a similar screen. Moreover, experimentally testing resistance requires information on particular small molecules that are often missing from COSMIC, and those for which these data were available were already part of our (limited) source of resistance variants for training the method.

Our aim was to devise minimal, inexpensive and rapid experiments that might catch whether a kinase was showing highly elevated activity, without engaging in expensive and time-consuming protocols. For each variant, we thus transfected T-REx-293 cells with tetracycline-compatible pDest30 vectors containing the gene of interest (wild type or mutant). After 24 h of tetracycline induction, we extracted RNA and assessed gene expression for biological replicates via microarrays (Additional file 1: Fig. S10). We compared induced cells to their transfected and non-induced counterparts and induced mutants to induce wild-type kinase. While T-REx-293 cells should not be used to investigate particular diseases (e.g. lymphoma), we used this standardised cell line to detect changes in kinase activity. For PIM1, we additionally used commercially available phospho-antibodies to assess the phosphorylation levels of key sites targeted by the kinases. For CHEK2, we also assessed sensitivity to ROS-induced DNA damage, and for MAP2K3, we investigated mitochondrial activity using the Mitotracker dye.

We saw little or no signal from our methods from the predicted deactivating variants, as might be expected given the traditional difficulties in establishing a loss of function. However, for one, the high expression similarity between the uncharacterised (predicted either resistance or deactivating) MAP2K1 p.Val211Asp and the known deactivating p.Lys97Ala suggests a loss of activity, particularly as the known activating variant p.Gln56Pro looks very different (Additional file 1: Fig. S8; Additional file 5: Table S27). For three of the four predicted activating variants, we saw clear differences to wild type that we discuss in the next sections. Activating variants are of particular interest in that they can immediately suggest treatment by way of specific inhibitors. These (and resistance variants) are thus of the clearest clinical relevance.

#### The PIM1 p.Ser97Asn lymphoma variant leads to increased or constitutive activation

**Fig. 4:**
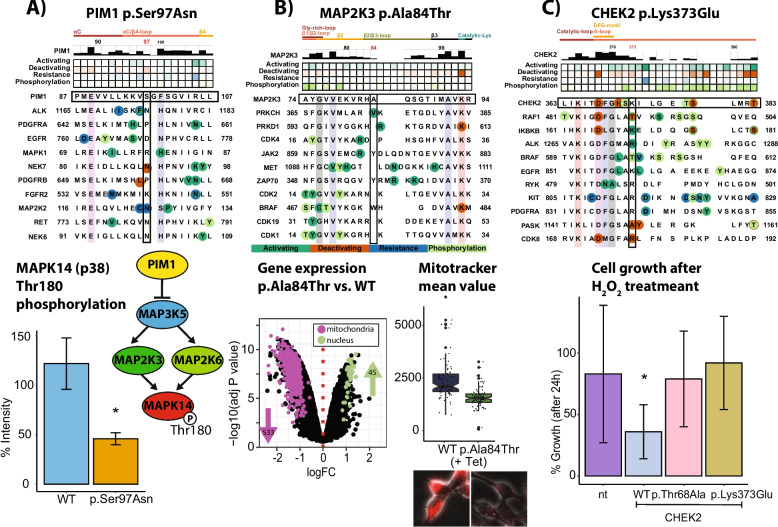
Experimental interrogation of selected predictions. **A** Top: alignment of selected kinases around PIM1 Ser97 with positions marked if they harbour known activating (green), deactivating (red) or resistance (blue) mutations or if they are known phosphosites (light green). Bottom: Light signal depletion measured ⍺-MAPK14/p38 Thr180 in cells transfected with PIM1 Ser97Asn compared to PIM1 wild type after 24 h of tetracycline induction (*n* = 4) based on the PIM1 dependent ASK1/MAP3K5 signalling pathway. **B** Top: alignment of selected kinases around MAP2K3 Ala84 with positions marked as in Fig. 3. Bottom left: volcano plot showing gene-expression changes when comparing T-REx-293 cells transfected with MAP2K3/p.Ala84Thr versus MAP2K3 wild type after 24 h of tetracycline induction. We only considered genes with 5% FDR (Benjamini-Hochberg) and a log2FC of at least 1 (*n* = 4). Bottom right: Plot showing how Mitotracker light signal in stained mitochondria was reduced in p.Ala84Thr compared to MAP2K3 WT overexpressing cells, with two exemplar images shown beneath. **C** Top: alignment of selected kinases around CHEK2 Lys373 with positions marked as in Fig. 3. Bottom: Relative cell growth of T-REx-293 wild-type cells versus cells transfected with CHEK2 wild-type or Thr68Ala or Lys373Glu. Cells were kept under tetracycline induction and counted 24 h after treatment with 400 µM H_2_O_2_. *n* = 3

Ser97 in PIM1 lies in the C-helix (Fig. 4A top). Within COSMIC, Ser97 is the position in PIM1 with the most missense variants (97), of which 37 are p.Ser97Asn, and all in haematopoietic and lymphoid cancers [56]. This variant is predicted to be weakly activating by our models. The loss of Asn at the equivalent position in NEK7 (p.Asn90Lys, p.Asn90Arg and p.Asn90Ala) [57] leads to a strong reduction in its kinase activity, suggesting that an Asn might be favoured at this position. There are also two known activating variants N-terminal to this position in ALK (p.Phe1174Leu/Val) [58].

Overexpression of mutant PIM1 showed no significant expression differences between p.Ser97Asn and wild type after 24 h of induction. We additionally investigated the phospho-Thr180 levels of MAPK14/p38, a well-known protein downstream of PIM1 signalling. We detected a twofold depletion in phosphorylation of MAPK14/p38 Thr180 (via ɑ-p38/pThr180 antibody), which would be expected if PIM1 activity was elevated (Fig. 4A, bottom, Additional file 5: Tables S24-S25), as MAPK14/p38 activation is dependent on ASK1 activation, which PIM1 inhibits [59].

MYC overexpression via enhancer hijacking is the hallmark of several lymphoid cancers, particularly Burkitt Lymphoma [60]. PIM1 phosphorylation of MYC is known to enhance its stability [61]. There are thus good arguments for why increased PIM1 function would be expected in these tumours as either a primary or secondary driver event. PIM1 is thought to be intrinsically active (i.e. it does not require other factors to become active) [62]. It is possible that p.Ser97Asn is augmenting an already active enzyme in the context of these tumours, possibly to stabilise MYC. This raises the possibility that the other COSMIC PIM1 variants seen in these tumours and predicted to be activating by our method (e.g. p.Gly28Asp, p.Glu135Lys, p.Pro33Ser; see Fig. 3B) also increase its activity. Indeed, structural variants of MYC are largely absent in samples from a Follicular Lymphoma cohort where PIM1 variants are enriched [56]. All of this argues that p.Ser97Asn and likely many of the other variants are indeed increasing PIM1 enzymatic activity as a possible driver of lymphoid tumours. PIM1 is an established oncogene, though this is generally because of observed elevation of expression, for instance in prostate cancer [63]. Our findings and the growing number of missense observations argue that activating variants might also play a role in PIM1-mediated pathogenesis.

#### MAP2K3 p.Ala84Thr: a rare constitutively active driver in various cancers that greatly depletes mitochondrial gene expression

MAP2K3 (MEK3, MKK3) p.Ala84Thr is seen in 24 samples within COSMIC, with the most common (10 samples) from head and neck cancers [25] in respiratory and upper-digestive tract tumours and predicted to be activating. Ala84 lies in the ꞵ2/3 loop and is eight residues C-terminal of the conserved GxGxxG motif (Fig. 4B, top). Inspection of other kinases shows evidence of activating variants in the same region (Fig. 4B, top). For instance, PRKD1 (protein kinase Db1) has an activating variant at this position: p.Arg603His, seen in telangiectasia-ectodermal dysplasia-brachydactyly-cardiac anomaly syndrome shows constitutive catalytic activity [51]. Variants at positions two residues N-terminal to Ala84 lead to constitutive activation in JAK2 (p.Arg867Gln [64]) and CDK4 (p.Arg24Cys [65]), as do variants C-terminal of this position, such as MET p.Asn1100Tyr [66] or ZAP70 p.Lys362Glu [67]. In addition, several kinases have phosphorylation sites in this region (Fig. 4B, top). For example, the Arabidopsis kinase SnRk2 is autophosphorylated at a position equivalent to Ser-86 in MAP2K3 (Ser-43 [68]).

Over-expression of the mutant MAP2K3 showed a marked difference in gene expression compared to the wild-type. Specifically, only p.Ala84Thr (and not wild-type or other variants, Additional file 1: Fig. S8A) showed a drastic reduction (adj. *p*-value < 10^–20^, Additional file 1: Fig. S7B) in the expression of hundreds of mitochondrial genes (Fig. 4B, bottom; Additional file 1: Fig. S6B, 7A; Additional file 5: Tables S21–S23). Mitochondrial gene under-expression is seen when comparing the mutant to WT at 24 h or when comparing the mutant at 24 h to 0 h (nothing is significant when comparing WT at 24 h to 0 h apart from roughly fourfold overexpression of MAP2K3). This is strong support for this mutation activating MAP2K3 as several studies have shown that down-regulation produces the opposite effect. Deletion of this enzyme in mice leads to an increase in mitochondria number and function [69, 70] and hyaluronan-mediated suppression of MAP2K3 expression in human mesenchymal stem cells similarly led to an increase in mitochondrial number and membrane potential [71]. The fact that we are seeing the opposite behaviour and that we see no effect of the wild type supports the idea that this mutant has elevated kinase activity. Assessing mitochondrial activity using MitoTracker (Thermo Fisher) supported this finding, showing that the tetracycline-induced p.Ala84Thr had significantly lower activity than the un-induced (Additional file 1: Fig. S9, Additional file 5: Table S26). To our knowledge, no mitochondrial phenotype has to date been reported during the over-expression of MAP2K3. Though the true nature of this variant in the context of cancer might be different (i.e. in contrast to T-REx-293 cells), it has been proposed that general suppression of respiratory (i.e. mitochondrial) gene expression is seen in many cancer types [72].

Breast cancer patients showing elevated expression of MAP2K3 have worse survival rates, particularly triple-negative, and the kinase is proposed to be oncogenic in driving MYC in certain patients [73]. MAP2K3 p.Ala84Thr has previously been classified as benign in a large screen of cancer somatic and germline genomes [43], likely as the kinase itself did not stand out as a major driver across the pan-cancer set. This variant shows a suspiciously high frequency in gnomAD (though never homozygous making it possibly not viable in two copies [28]), though it is intriguingly absent from the 1000 Genomes dataset [74] (the gnomAD frequency would suggest over 300 counts in 1000 Genomes), making its population status somewhat unclear. Many samples for this variant in gnomAD are also marked as having failed the inbreeding coefficient filter. Moreover, p.Ala84Thr is one of only two of eleven variants with an allele frequency > 0.001 that were not filtered out completely (indeed p.Ala84Val is filtered out), which further questions the legitimacy of this record. Regardless, the gene-expression and MitoTracker results support the notion that this variant could lead to increased MAP2K3 activity.

#### The curious case of CHEK2 p.Lys373Glu

We predicted p.Lys373Glu as a putative activating variant in checkpoint kinase 2 (CHEK2; Fig. 4C, top). Through phosphorylation of numerous substrates, CHEK2 regulates cell cycle arrest, DNA repair and apoptosis upon DNA damage, thus acting as a tumour suppressor (e.g [75].). Most somatic or cancer-predisposition variants in this kinase have been shown to result in loss or decreased kinase activity (e.g [76, 77].).

Within COSMIC, this is the most common variant (present in 102 samples; the next is 47) seen in the large intestine, nervous system, kidney and other cancers. Lys373 lies three residues C-terminal of the conserved DFG motif. This region in other kinases contains a mixture of activating and deactivating variants. For example, the exact equivalent variant leads to constitutive or increased activity in IKBKB (p.Lys171Glu [78, 79]) and an Arg to Gln change in ALK is a known constitutively active mutation (p.Arg1275Gln [80]). In contrast, a gain of a Lysine at this position can be deactivating, such as seen in PASK (p.Ala1151Lys [81]). The loss of a positive charge at this position has also been observed to be deactivating, for example, in CDK8 (p.Arg178Gln) [82]. Deactivating variants are less similar; for example, p.His371Tyr two positions N-terminal in CHEK2 in breast cancer [83] and the equivalent N-terminal position p.Leu597Val in BRAF was shown to be activating [84].

There are thus good arguments for why this modification changing a lysine to glutamate might be activating in CHEK2 despite its role as a tumour suppressor. Running against this, activation of CHEK2 has recently been shown to confer resistance to oxaliplatin treatment in colorectal cancer [85] and overexpression of CHEK2 was linked to worse survival in adrenocortical carcinoma [86]. Intriguingly, CHEK2 p.Lys373Glu is strongly correlated with patients’ progression-free survival in high-grade serous ovarian carcinoma post-olaparib treatment [87]. Taken together, this might suggest that the outcome of CHEK2 signalling perturbation is tissue-specific.

We could see no gene expression difference between induced over-expressed CHEK2 p.Lys373Glu and the wild-type enzyme or a kinase-dead variant (CHEK2 p.Thr68Ala [88]). However, there is a pronounced difference between both variants and the CHEK2 WT when monitoring cell counts over a period of 72 h with periodic treatment with the DNA-damaging agent H2O2 (Fig. 4C, bottom; Additional file 5: Tables S19–S20). Both variants appear to show a perturbation of the enzyme in that they have higher cell numbers than the wild type, resulting in increased apoptosis resistance. Thus, it is clear, as has been shown previously [89], that p.Lys373Glu is likely deactivating in T-REx-293 cells.

Inspection of CHEK2 structures offers some possible insights as to why this predicted activating variant is, in fact, deactivating. Lys-373 lies at the dimer interface in both dimeric forms of the enzyme [90, 91], and is thought to play a key role in CHEK2 activation [91], in a manner that likely differs from most other kinases. The context of CHEK2 activation (not considered in our predictor) is thus likely why this was wrongly predicted. We are currently experimenting with adding additional features related to dimer contacts, though this requires a more complete set of reliable homo/hetero-dimer structures than are currently available.

## Discussion

Despite great evolutionary diversity, there are still clear common trends within kinases about how variants affect them. For instance, just 14 alignment positions capture roughly 33% (494 out of 1501) of known functional sites. The simple presence of a variant at a position, while often predictive, is not sufficient, however, particularly for variants occurring at phosphosites where the charge on the mutated amino acid (negative or not) determines the likely effect (activating or deactivating).

A major result of this work is the simple overlay of carefully annotated positional information on variants and functions in the context of a well-constructed multiple-sequence alignment (Fig. 1). The ability to exploit these data via machine learning to predict whether variants are activating, deactivating or resistance-related should prove useful to those wishing to interrogate kinase variants in the context of diseases and particularly cancers. This is an important addition to the field because we are not predicting whether a kinase variant is pathogenic, but rather the functional outcome of kinase variants. This is useful as activating kinase variants are often a lot more subtle (and harder to predict in general) than deactivating kinase variants, where tools such as AlphaMissense might miss them. Indeed, our two experimentally confirmed activating variants (MAP2K3 p.Ala84Thr and PIM1 p.Ser97Asn) are predicted as neutral by most of the methods we investigated here [5–7]. Application of our predictor to the entire sets of somatic and hereditary disease variants affecting kinase domain positions both provided additional evidence for the method’s efficacy and showed that variants for particular kinases as a whole tend to be predicted as expected (e.g. oncogenes have mostly activating variants; tumour suppressors deactivating). While this is of course a confirmation of what medical professionals would have expected after using predictors of pathogenicity on such variants, our tool provides novel information by predicting whether this ‘pathogenic’ variant is indeed increasing kinase activity (‘activating’), highlighting that our tool is not meant to replace existing predictors of pathogenicity but rather to be used alongside them. Given predictions for variants of interest, the comparatively simple tests we performed (i.e. via gene expression, phosphoantibodies or comparatively rapid cell biology tests) demonstrate that it is possible to also couple the computational analysis (i.e. using our predictor alongside other general predictors of pathogenicity) to experiments within the time frame of diagnostic and treatment decisions (i.e. 2–3 months, Additional file 5: Table S27).

Our two new likely activating variants (in PIM1 and MAP2K3) have very low sample counts in current cancer datasets. Indeed, considering only tumour-derived, confirmed somatic variants the counts are only 11 for PIM1 p.Ser97Asn and six for MAP2K3 p.Ala84Thr. This suggests that there might be many others previously overlooked (e.g. in Additional file 4: Table S16) owing to their rarity, but which could nevertheless be informative in the context of the particular patients harbouring them. Regardless of frequency, the ability to identify rapidly and characterise such variants of unknown significance can have immediate consequences, particularly for protein kinases. Knowing that a kinase contains an activating or resistance variant can immediately suggest changes to treatment regimens that are more appropriate to the specific patient. For 293 out of 505 kinases, there are drugs already on the market; for an additional 7, there are compounds in development and earlier candidates likely for many of the remainder (data from PKIDB [92]; retrieved 30 June 2023). For example, both PIM1 and MAP2K3 lack clinically approved inhibitors, but both have compounds in different stages of development [63, 93]. We thus believe that approaches like the one provided here, together with the exponential growth in sequencing and an increasing arsenal of modulation strategies, will assist researchers to rapidly assess novel variants of unknown significance and help medical professionals make personalised medicine a reality.

## Conclusions

We developed and evaluated a curated, kinase-focused framework to predict the functional consequences of missense variants, distinguishing activating, deactivating, resistance, and neutral classes. By integrating sequence, evolutionary, structural, and post-translational modification features, our gradient boosting models achieved high accuracy, and their predictions aligned with the expected enrichments of activating mutations in cancer and deactivating ones in hereditary disease. Experimental validation for two of three selected variants supported the predicted gain-of-function effects. The accompanying web application (activark.russelllab.org) provides open access to the predictors and underlying data, enabling rapid assessment of kinase-domain variants. Together, these resources extend pathogenicity prediction beyond binary classification toward mechanistic interpretation, helping to prioritise clinically actionable kinase variants and support precision medicine.

## Supplementary Information

Additional file 1. Supplementary Figures S1-S10.

Additional file 2. Supplementary Tables S1-S8. 

Additional file 3. Supplementary Tables S9-S15. 

Additional file 4. Supplementary Tables S16-S18.

Additional file 5. Supplementary Tables S19-S27. 

## Data Availability

The datasets curated and used to develop the machine learning models are available online [22]. Microarray data were deposited in the Gene Expression Omnibus (GSE232293) [36]. Additional analyses supporting the conclusions of this study have been supplied as Supplementary Information. Machine learning predictions described in this study can be carried out at activark.russelllab.org. The code is available online at GitHub [94] and is released under the GNU General Public License v.3.
