# Supplementary material for: Discriminating activating, deactivating and resistance variants in protein kinases
Source: Genome Med. 2025 Oct 28;17:133. doi: 10.1186/s13073-025-01564-z (PMC12570665; doi:10.1186/s13073-025-01564-z)
Supplement: Supplementary file 1 — Additional file 1. Supplementary Figures S1-S10. [file 13073_2025_1564_MOESM1_ESM.pdf]

Supplementary Information: Discriminating activating, deactivating and resistance variants in protein kinases

Gurdeep Singh (1)*, Torsten Schmenger (1)*, Juan Carlos Gonzalez-Sanchez* (1), Anastasiia Kutkina (1), Nina Bremec (1), Gaurav D Diwan (1), Pablo Mozas (2,3), Cristina López (3), Reiner Siebert (4), Rocio Sotillo (5), Robert B Russell (1)+

1. BioQuant & Heidelberg University Biochemistry Center, Im Neuenheimer Feld 267, 69121 Heidelberg, Germany

2. Department of Hematology, Hospital Clínic de Barcelona, Barcelona, Spain

3. Fundació de Recerca Clínic Barcelona-Institut d'Investigacions Biomèdiques August Pi i Sunyer (FRCB-IDIBAPS); Departament de Fonaments Clínics, Facultat de Medicina i Ciències de la Salut, Universitat de Barcelona, Barcelona, Spain; Centro de Investigación Biomédica en Red de Cáncer (CIBERONC), Madrid, Spain.

4. Institute of Human Genetics, Ulm University and Ulm University Medical Center, Ulm, 89081, Germany.

5. Division of Molecular Thoracic Oncology, German Cancer Research Center (DKFZ), Translational Lung Research Center (TLRC), Member of the German Center for Lung Research (DZL), 69120, Heidelberg, Germany.

* Equal contribution

+Correspondence:

robert.russell@bioquant.uni-heidelberg.de, Tel: +49 6221 54 51 362

Figure S1 - Summary of known functional variants in human kinases

A) Plot showing the source and distribution of variants known to be activating (lead to constitutive activation or increase in kinase activity), deactivating (lead to loss or decrease in kinase activity) and resistance-causing. B) Plot showing the distribution of known functional variants at most conserved (in human kinases and orthologs) and post-translational modification (PTM) sites. C) Plot showing the distribution of known functional variants across the N-, C-lobes and activation loop of the canonical kinase domain. D) Plot showing counts of different types of known functional variants in the canonical kinase domain in the curated dataset. E) Plot showing most mutated sites (with a count of at least 10 in our dataset) in the canonical kinase domain. Positions correspond to domain locations as indicated in Fig S2.

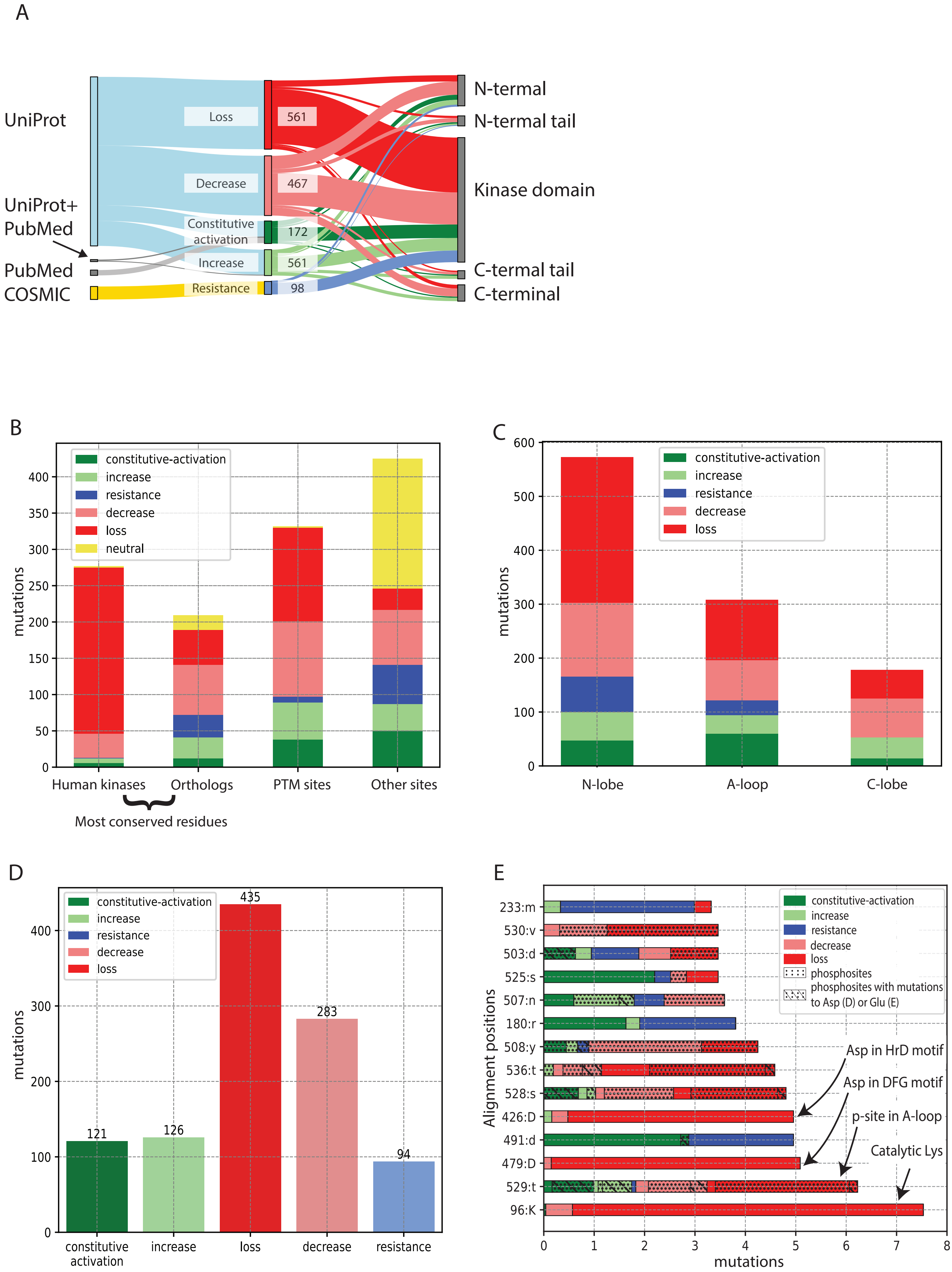
A
UniProt
UniProt+
PubMed
PubMed
COSMIC
Loss
Decrease
Constitutive
activation
Increase
Resistance
561
467
172
561
98
N-termal
N-termal tail
Kinase domain
C-termal tail
C-terminal
B
constitutive-activation
increase
resistance
decrease
loss
neutral
mutations
Human kinases
Orthologs
PTM sites
Other sites
Most conserved residues
C
N-lobe
A-loop
C-lobe
D
121
126
435
283
94
constitutive
activation
increase
loss
decrease
resistance
E
Alignment positions
233:m
530:v
503:d
525:s
507:n
180:r
508:y
536:t
528:s
426:D
491:d
479:D
529:t
96:K
phosphosites
phosphosites with mutations
to Asp (D) or Glu (E)
Asp in HrD motif
Asp in DFG motif
p-site in A-loop
Catalytic Lys

Figure S2 - Distribution of known variants in the two major kinase classes

A) Plot showing the distribution of known functional and neutral variants in human Serine/Threonine kinases (Pfam: Pkinase). Each bar corresponds to a position in the alignment constructed using human kinases. Variants are coloured based on their likely effect on the kinase activity: constitutively-activated (dark green), increase (light green), loss (red), decrease (orange), and neutral (yellow). Resistance-causing variants are coloured blue. The total number of variants at an alignment position is log normalised (base 2) and divided based on the proportion of each variant type. Most conserved sites in the kinase domain are annotated with text and arrows. Phosphorylation and Ubiquitination sites are highlighted in cyan and grey colours at the base of the bar. Structure in the centre refers to a canonical kinase domain and its secondary structure elements (INSR kinase, PDB ID: 1GAG). B) as in A but for Tyrosine kinases (Pfam: PK_Tyr_Ser/Thr).

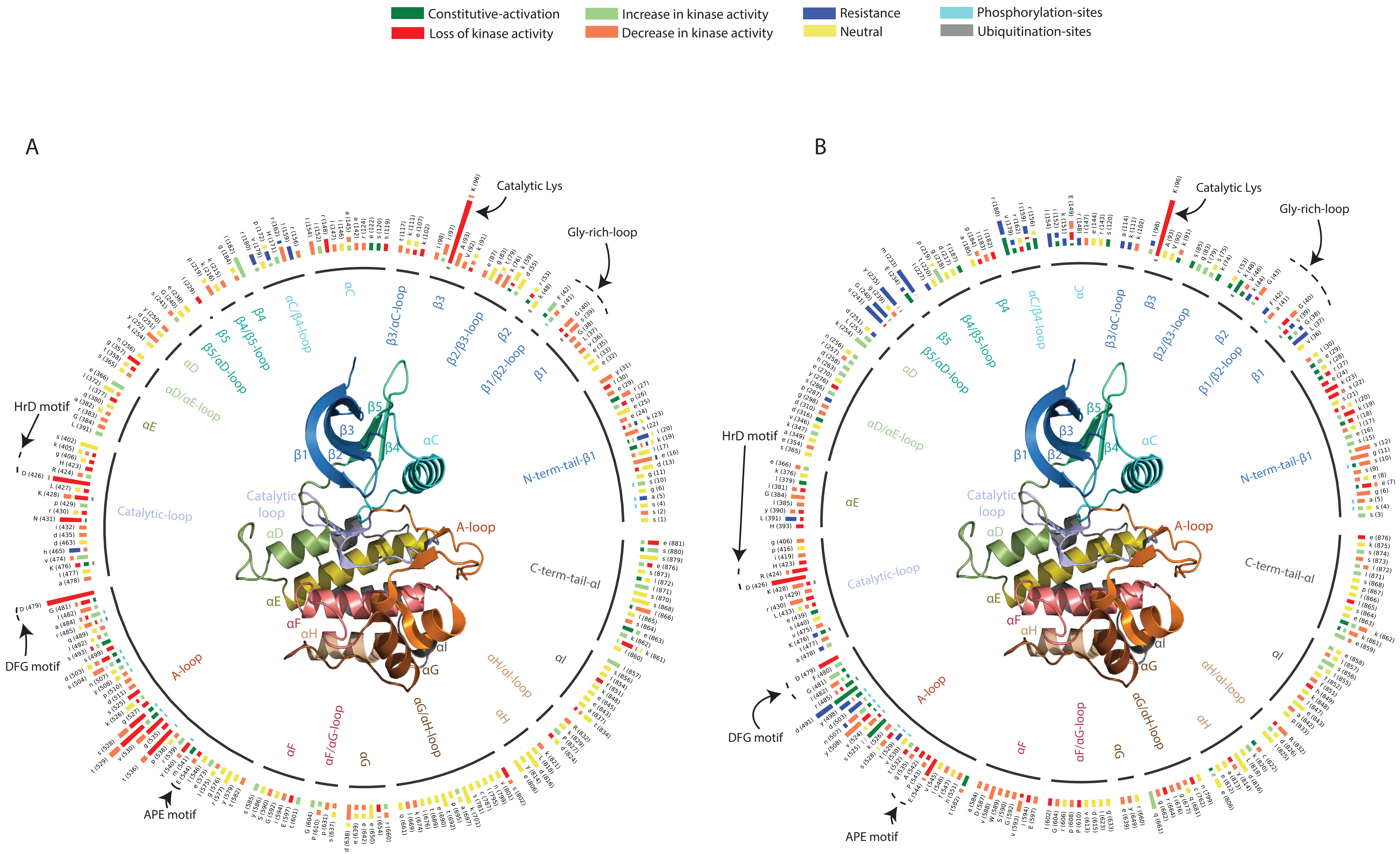

Constitutive-activation
Loss of kinase activity
Increase in kinase activity
Decrease in kinase activity
Resistance
Neutral
Phosphorylation-sites
Ubiquitination-sites
A
Catalytic Lys
Gly-rich-loop
HrD motif
DFG motif
APE motif
N-term-tail-β1
β1
β1/β2-loop
β2
β2/β3-loop
β3
β3/αC-loop
αC
αC/β4-loop
β4
β4/β5-loop
β5
β5/αD-loop
αD
αD/αE-loop
αE
Catalytic-loop
A-loop
αF
αF/αG-loop
αG
αG/αH-loop
αH
αH/αI-loop
αI
C-term-tail-αI
Catalytic loop
B
Catalytic Lys
Gly-rich-loop
HrD motif
DFG motif
APE motif

Figure S3 - Performance of the predictors during the training phase

A-D) Plots showing Receiver Operator Characteristic (ROC) curves of the 10-fold cross-validation (training) results of the predictor activating vs. deactivating (A); activating vs deactivating/neutral (B); deactivating vs activating/neutral (C); resistance vs neutral (D). Note: for the three-way activating vs deactivating vs neutral predictor (B & C), we computed the AUC for each class (activating/deactivating) against the rest. (E) Confusion matrices and precision-recall curves for our three predictors (from top to bottom) "Activating vs Deactivating", "Activating vs Deactivating vs Neutral" and "Resistance vs Neutral", using Gradient Boosting (left) or Random Forest (right) as algorithm.

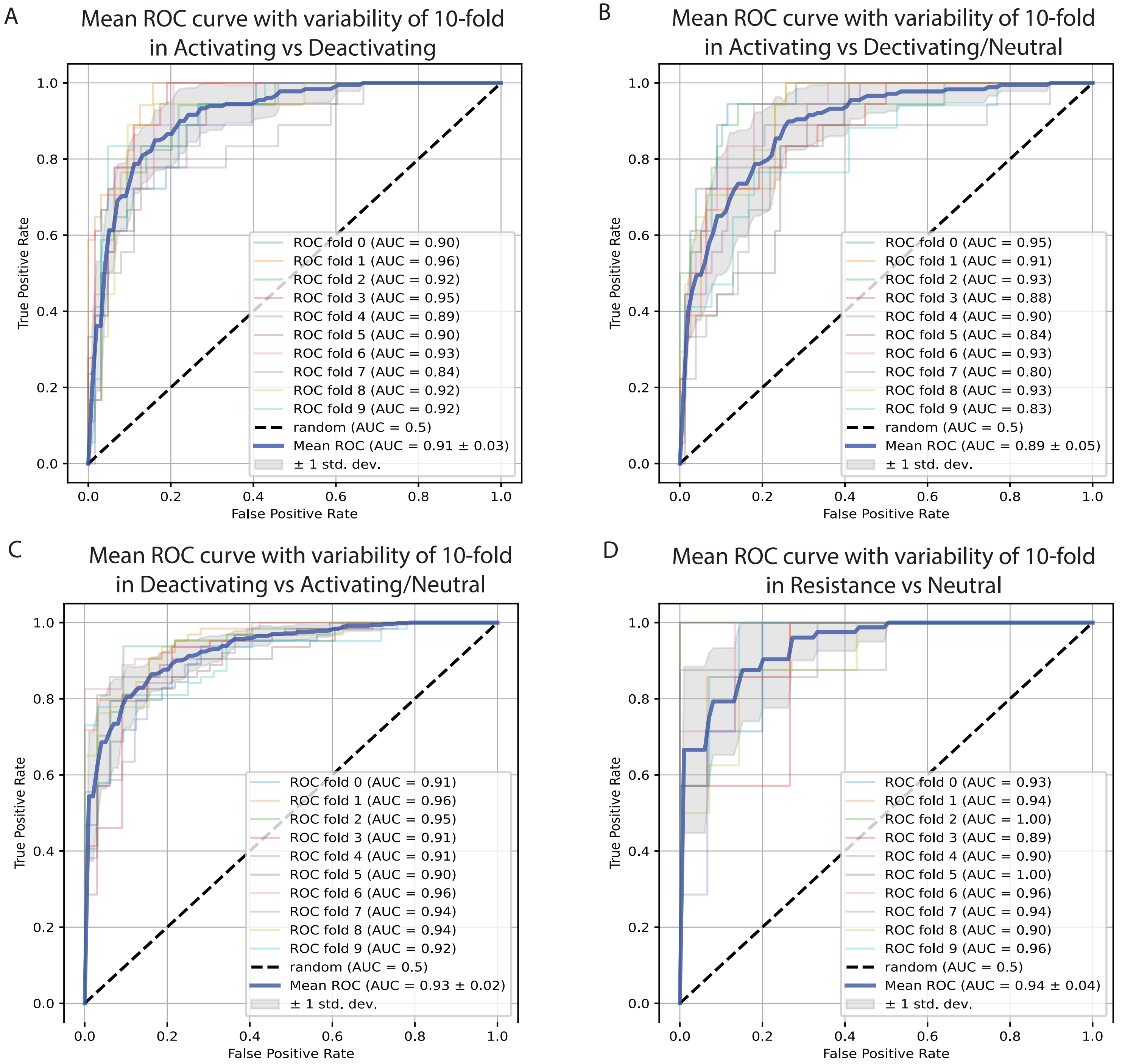
A
Mean ROC curve with variability of 10-fold in Activating vs Deactivating
True Positive Rate
False Positive Rate
ROC fold 0 (AUC = 0.90)
ROC fold 1 (AUC = 0.96)
ROC fold 2 (AUC = 0.92)
ROC fold 3 (AUC = 0.95)
ROC fold 4 (AUC = 0.89)
ROC fold 5 (AUC = 0.90)
ROC fold 6 (AUC = 0.93)
ROC fold 7 (AUC = 0.84)
ROC fold 8 (AUC = 0.92)
ROC fold 9 (AUC = 0.92)
random (AUC = 0.5)
Mean ROC (AUC = 0.91 ± 0.03)
± 1 std. dev.
B
Mean ROC curve with variability of 10-fold in Activating vs Dectivating/Neutral
True Positive Rate
False Positive Rate
ROC fold 0 (AUC = 0.95)
ROC fold 1 (AUC = 0.91)
ROC fold 2 (AUC = 0.93)
ROC fold 3 (AUC = 0.88)
ROC fold 4 (AUC = 0.90)
ROC fold 5 (AUC = 0.84)
ROC fold 6 (AUC = 0.93)
ROC fold 7 (AUC = 0.80)
ROC fold 8 (AUC = 0.93)
ROC fold 9 (AUC = 0.83)
random (AUC = 0.5)
Mean ROC (AUC = 0.89 ± 0.05)
± 1 std. dev.
C
Mean ROC curve with variability of 10-fold in Deactivating vs Activating/Neutral
True Positive Rate
False Positive Rate
ROC fold 0 (AUC = 0.91)
ROC fold 1 (AUC = 0.96)
ROC fold 2 (AUC = 0.95)
ROC fold 3 (AUC = 0.91)
ROC fold 4 (AUC = 0.91)
ROC fold 5 (AUC = 0.90)
ROC fold 6 (AUC = 0.96)
ROC fold 7 (AUC = 0.94)
ROC fold 8 (AUC = 0.94)
ROC fold 9 (AUC = 0.92)
random (AUC = 0.5)
Mean ROC (AUC = 0.93 ± 0.02)
± 1 std. dev.
D
Mean ROC curve with variability of 10-fold in Resistance vs Neutral
True Positive Rate
False Positive Rate
ROC fold 0 (AUC = 0.93)
ROC fold 1 (AUC = 0.94)
ROC fold 2 (AUC = 1.00)
ROC fold 3 (AUC = 0.89)
ROC fold 4 (AUC = 0.90)
ROC fold 5 (AUC = 1.00)
ROC fold 6 (AUC = 0.96)
ROC fold 7 (AUC = 0.94)
ROC fold 8 (AUC = 0.90)
ROC fold 9 (AUC = 0.96)
random (AUC = 0.5)
Mean ROC (AUC = 0.94 ± 0.04)
± 1 std. dev.

E

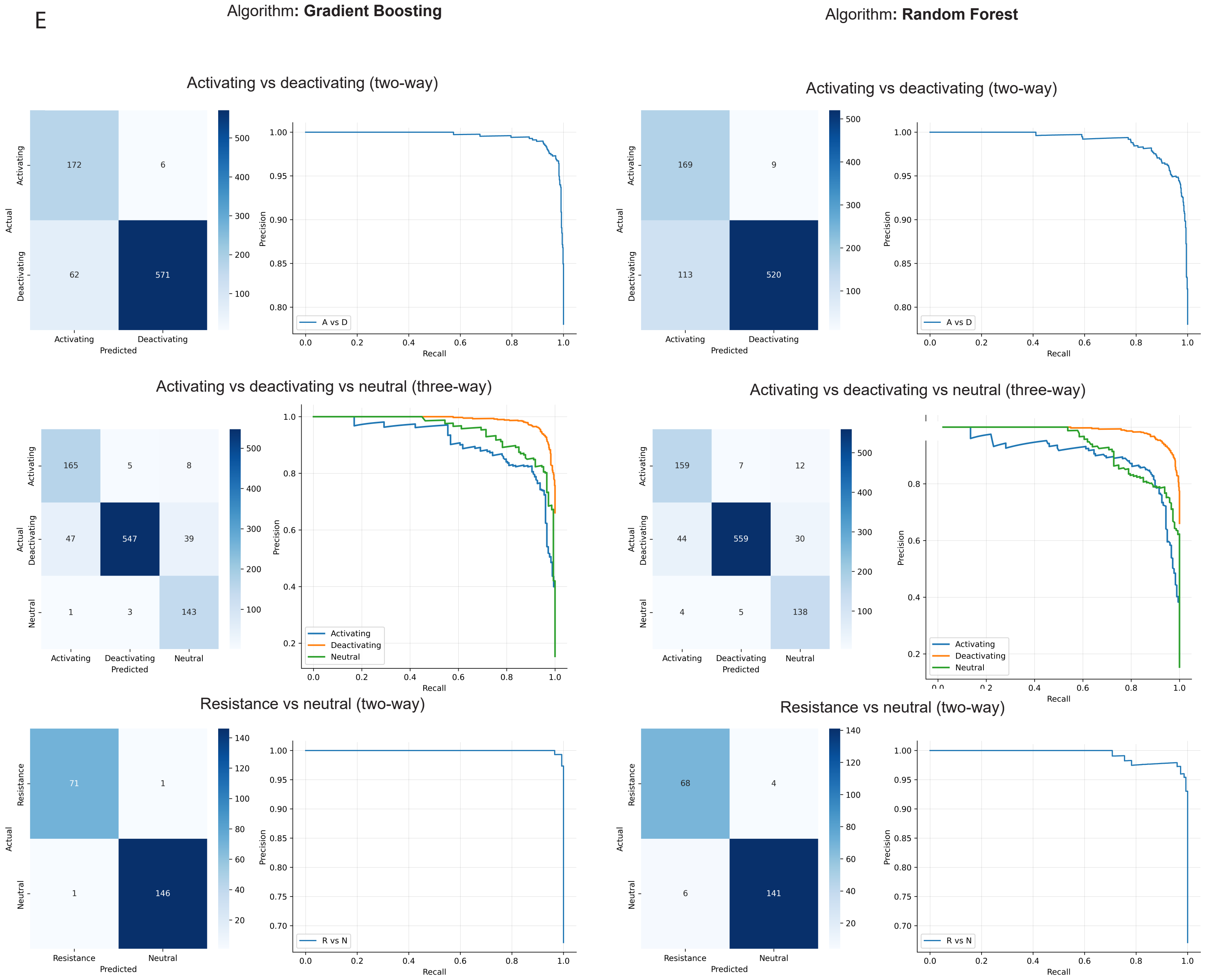

Algorithm: **Gradient Boosting**
Algorithm: **Random Forest**
Activating vs deactivating (two-way)
172
6
62
571
Activating
Deactivating
Actual
Predicted
Precision
Recall
A vs D
169
9
113
520
Activating vs deactivating vs neutral (three-way)
165
5
8
47
547
39
1
3
143
Neutral
159
7
12
44
559
30
4
5
138
Resistance vs neutral (two-way)
71
1
1
146
Resistance
R vs N
68
4
6
141

Figure S4 - Performance of predictors and other methods on the test set

ROC curves showing the performance of different predictors/ML approaches/methods on the test dataset (A = activating/increase; D = loss/decrease, R=resistance, N=neutral). A) GBC performance of activating vs neutral (values from three-way ADN predictor) B) GBC performance of a deactivating vs neutral (values from three-way ADN predictor) C) GBC performance of a binary neutral vs random predictor D) GBC performance (2-way and 3-way predictors) testing against different discriminations with AUC values given in the legend. E) for A but with variants from the test set removed if they occurred at the same protein and position as one in the training set (i.e. but with a different mutant residue). F) As for A but for the Random Forest predictor. G) Treating A, D & R as one type (i.e. "pathogenic") in order to compare to three known predictors of pathogenicity (AlphaMissense, PMUT, PolyPhen2 HVAR). Note that for D, E & G no fair comparison is possible to the other methods as they do not distinguish A, D & R from each other.

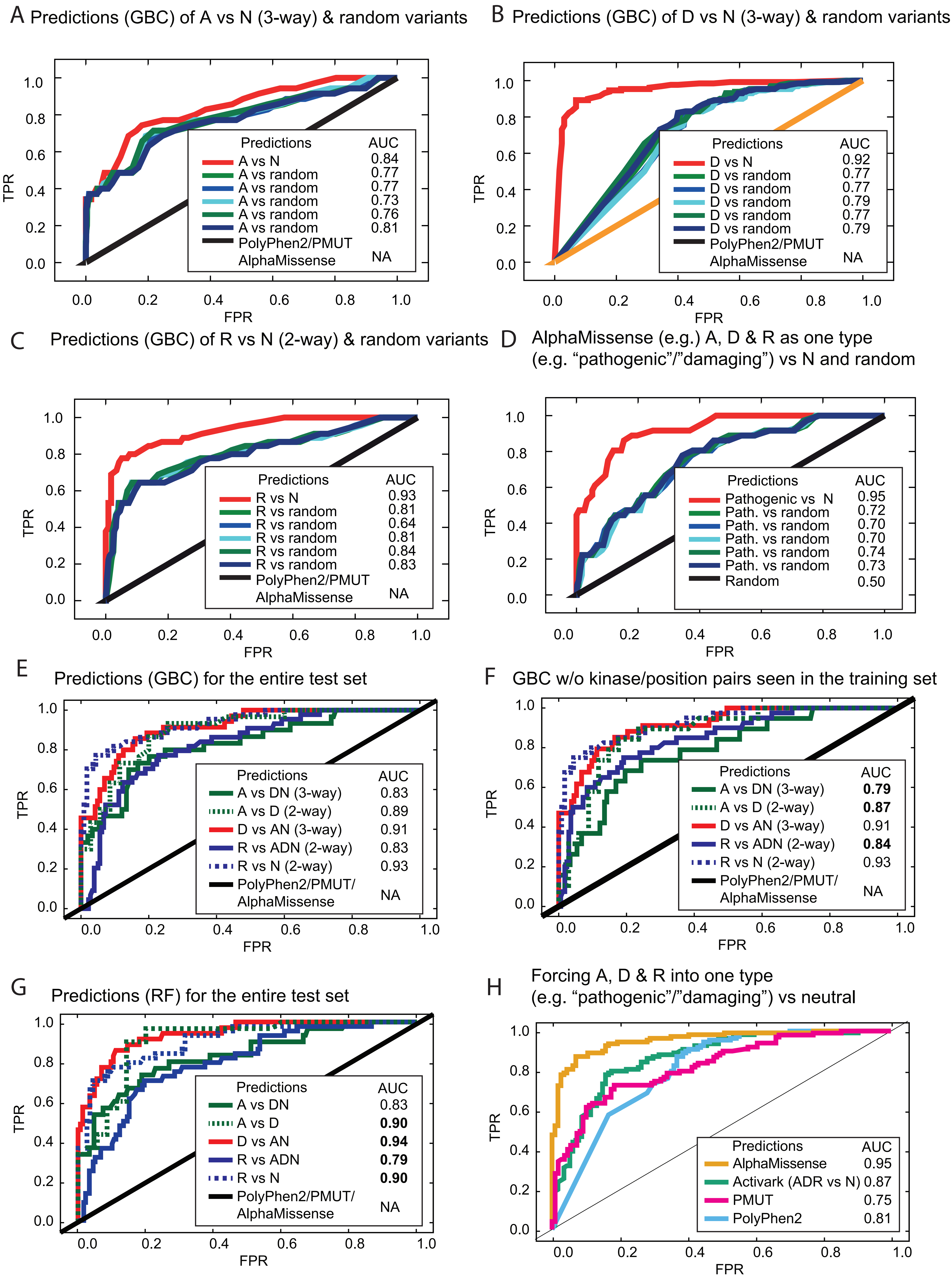
A Predictions (GBC) of A vs N (3-way) & random variants
Predictions AUC
A vs N 0.84
A vs random 0.77
A vs random 0.77
A vs random 0.73
A vs random 0.76
A vs random 0.81
PolyPhen2/PMUT
AlphaMissense NA
TPR
FPR
B Predictions (GBC) of D vs N (3-way) & random variants
Predictions AUC
D vs N 0.92
D vs random 0.77
D vs random 0.77
D vs random 0.79
D vs random 0.77
D vs random 0.79
PolyPhen2/PMUT
AlphaMissense NA
C Predictions (GBC) of R vs N (2-way) & random variants
Predictions AUC
R vs N 0.93
R vs random 0.81
R vs random 0.64
R vs random 0.81
R vs random 0.84
R vs random 0.83
PolyPhen2/PMUT
AlphaMissense NA
D AlphaMissense (e.g.) A, D & R as one type
(e.g. "pathogenic"/"damaging") vs N and random
Predictions AUC
Pathogenic vs N 0.95
Path. vs random 0.72
Path. vs random 0.70
Path. vs random 0.70
Path. vs random 0.74
Path. vs random 0.73
Random 0.50
E Predictions (GBC) for the entire test set
Predictions AUC
A vs DN (3-way) 0.83
A vs D (2-way) 0.89
D vs AN (3-way) 0.91
R vs ADN (2-way) 0.83
R vs N (2-way) 0.93
PolyPhen2/PMUT/
AlphaMissense NA
F GBC w/o kinase/position pairs seen in the training set
Predictions AUC
A vs DN (3-way) 0.79
A vs D (2-way) 0.87
D vs AN (3-way) 0.91
R vs ADN (2-way) 0.84
R vs N (2-way) 0.93
PolyPhen2/PMUT/
AlphaMissense NA
G Predictions (RF) for the entire test set
Predictions AUC
A vs DN 0.83
A vs D 0.90
D vs AN 0.94
R vs ADN 0.79
R vs N 0.90
PolyPhen2/PMUT/
AlphaMissense NA
H Forcing A, D & R into one type
(e.g. "pathogenic"/"damaging") vs neutral
Predictions AUC
AlphaMissense 0.95
Activark (ADR vs N) 0.87
PMUT 0.75
PolyPhen2 0.81

Figure S5 - Feature importance

A-C) Plots showing feature importance calculated individually for each of the 3 predictors: neutral vs deactivating vs activating(A); activating vs deactivating (B); resistance vs neutral (C). Each cell corresponds to the feature importance value. The sum of all feature importance values for a predictor is 1. Features with high values have higher colour gradients and thus are likely to have a more significant influence on the predictions.

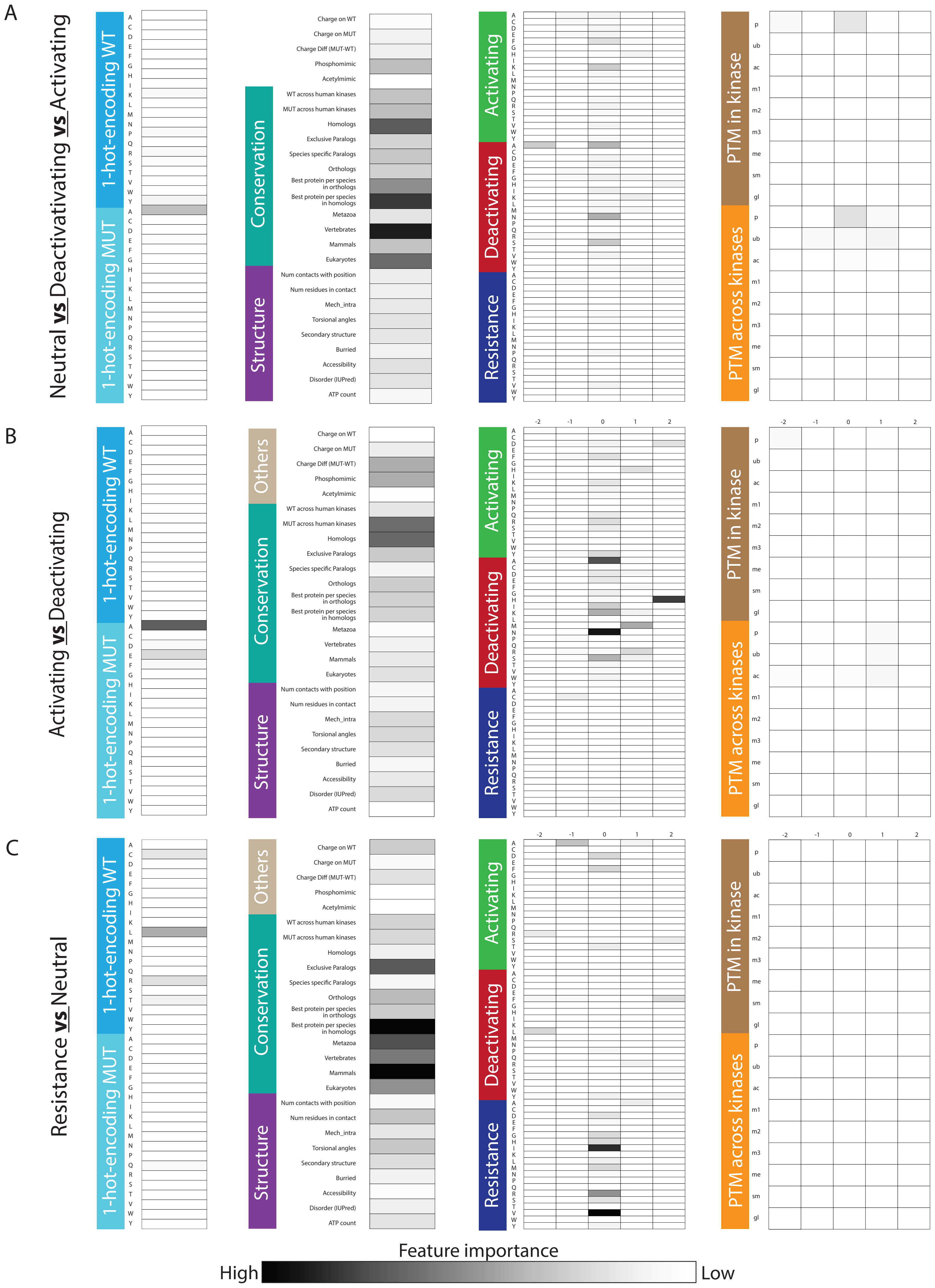

A
Neutral vs Deactivativating vs Activating
1-hot-encoding WT
1-hot-encoding MUT
Conservation
Structure
Charge on WT
Charge on MUT
Charge Diff (MUT-WT)
Phosphomimic
Acetylmimic
WT across human kinases
MUT across human kinases
Homologs
Exclusive Paralogs
Species specific Paralogs
Orthologs
Best protein per species in orthologs
Best protein per species in homologs
Metazoa
Vertebrates
Mammals
Eukaryotes
Num contacts with position
Num residues in contact
Mech_intra
Torsional angles
Secondary structure
Burried
Accessibility
Disorder (IUPred)
ATP count
Activating
Deactivating
Resistance
PTM in kinase
PTM across kinases
p
ub
ac
m1
m2
m3
me
sm
gl
B
Activating vs Deactivating
Others
-2
-1
0
1
2
C
Resistance vs Neutral
Feature importance
High
Low

Figure S6 - Gene expression control volcano plots

Gene expression volcano plots showing different control contrasts. All are between pairs of induced (+tet) or not-induced (-tet) HEK293T cells transfected with particular constructs. A) top: PIM1 p.Ser97Asn +tet vs PIM1 WT +tet, n=4. centre: PIM1 p.Ser97Asn +tet vs -tet, n=4. bottom: PIM1 WT +tet vs -tet, n=4. B) MAP2K3 p.Ala83Thr +tet vs MAP2K3 WT +tet, n=3. centre: MAP2K3 p.Ala83Thr +tet vs –tet, n=3. bottom: MAP2K3 WT +tet vs -tet, n=4. C) top: CHEK2 p.Lys373Glu (+tet) CHEK2 WT (+tet), n=3. centre: CHEK2 p.Lys373Glu (+tet) vs (-tet) control, n=3. bottom: CHEK2 WT (+tet) vs. (-tet) control, n=4.

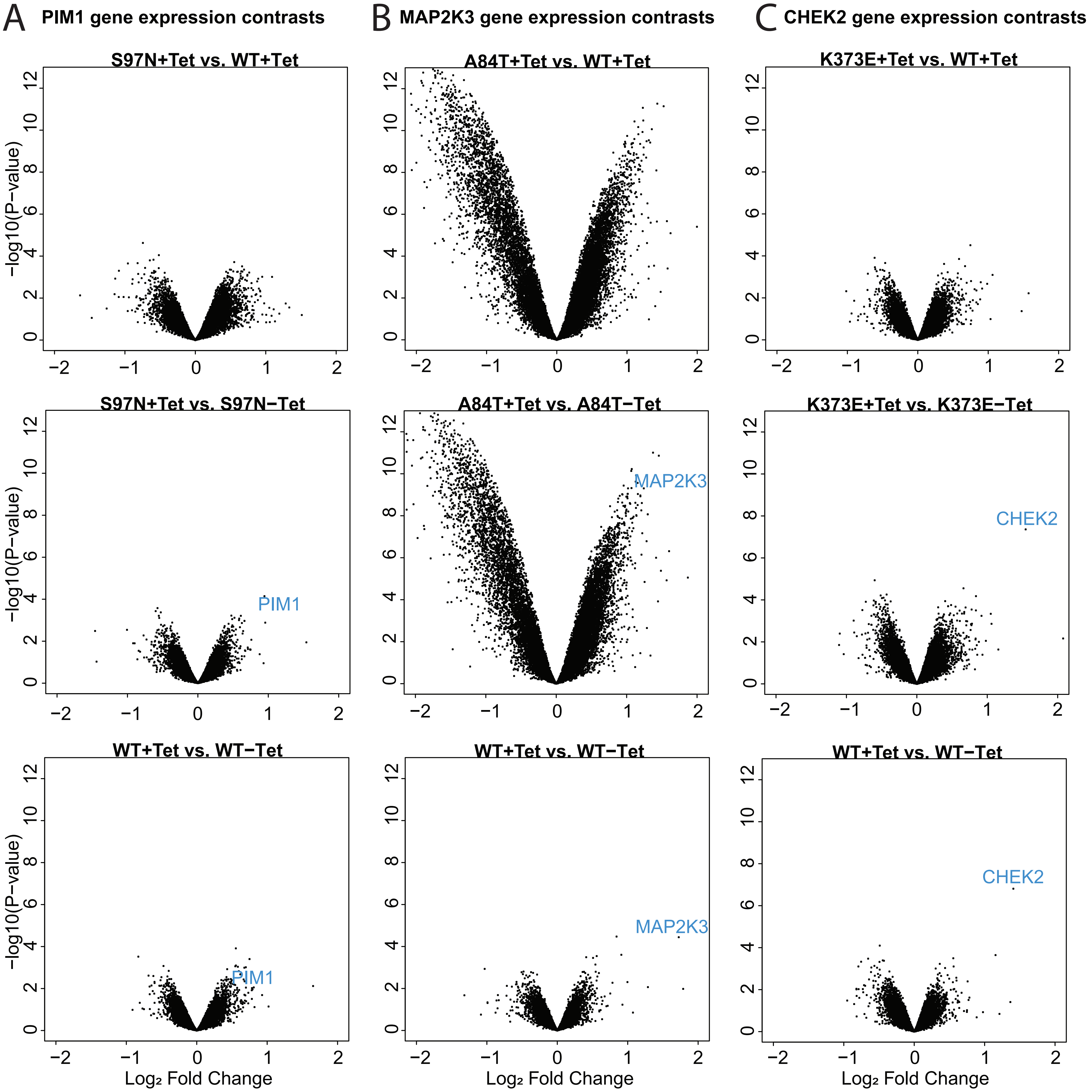
A PIM1 gene expression contrasts
B MAP2K3 gene expression contrasts
C CHEK2 gene expression contrasts
S97N+Tet vs. WT+Tet
A84T+Tet vs. WT+Tet
K373E+Tet vs. WT+Tet
S97N+Tet vs. S97N−Tet
A84T+Tet vs. A84T−Tet
K373E+Tet vs. K373E−Tet
WT+Tet vs. WT−Tet
PIM1
MAP2K3
CHEK2
−log10(P−value)
Log₂ Fold Change

Figure S7 - Differential gene expression

A) Heatmap showing differential gene expression between T-REx-293 cells transfected with MAP2K3 wild type (WT) or Ala84Thr (A84T) in the presence or absence (-tet, controls) of tetracycline induction. Clustering differentiates between cells overexpressing Ala84Thr and cells overexpressing MAP2K3 wild-type or control cells. Red and yellow shadings represent higher and lower expression levels, respectively. Genes associated with mitochondrial function are highlighted (y-axis), n=4. B) The functional annotation of differentially regulated genes was performed via the g:Profiler web server using default settings. We show the 10 most significant terms from up- (green) and downregulated (purple) genes.

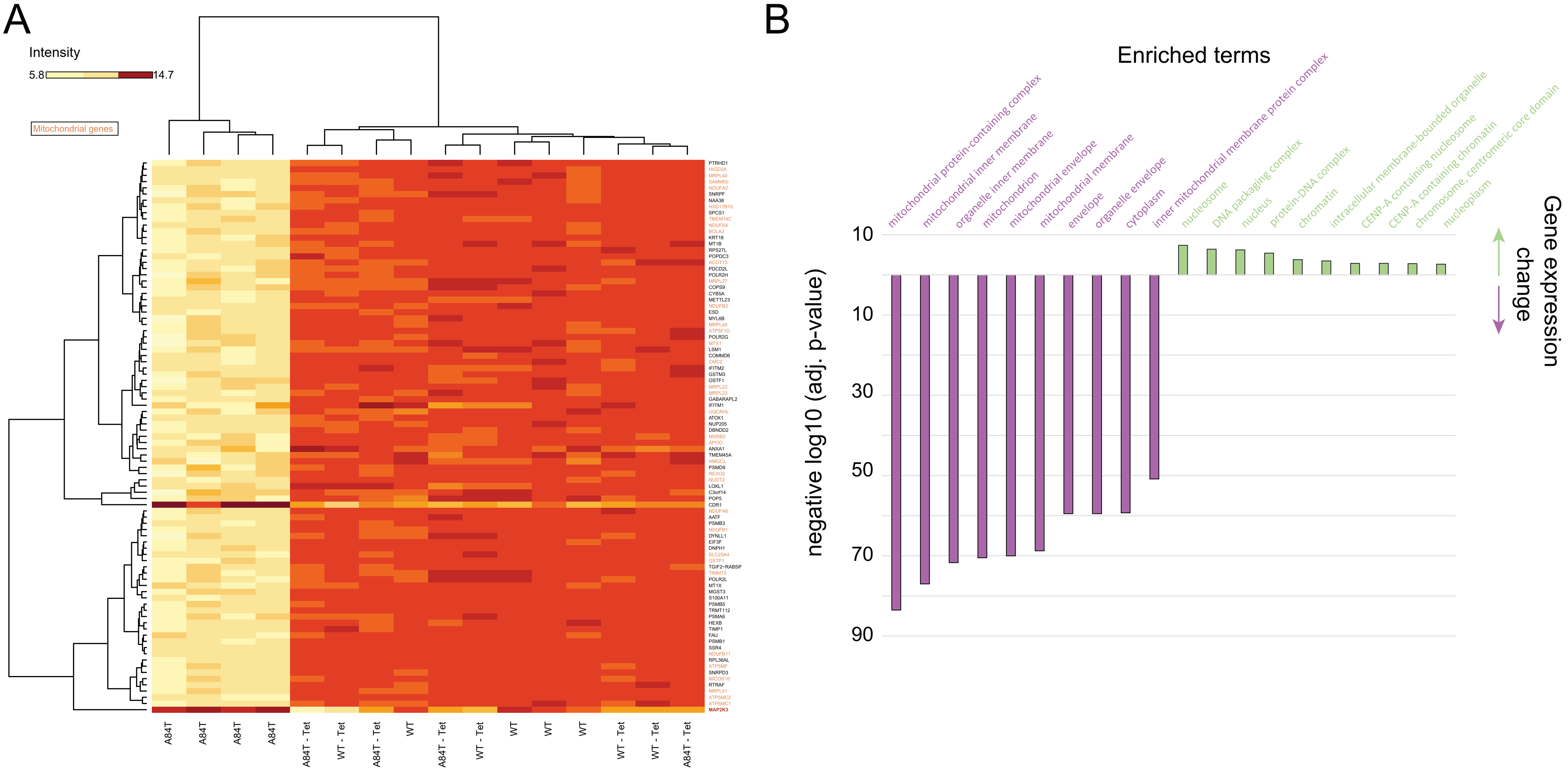
A
Intensity
5.8
14.7
Mitochondrial genes
PTRHD1
HIGD2A
MRPL40
SAMM50
NDUFA2
SNRPF
NAA38
HSD17B10
SPCS1
TMEM14C
NDUFS4
BOLA3
KRT18
MT1B
RPS27L
POPDC3
ACOT13
PDCD2L
POLR2H
MRPL27
COPS9
CYB5A
METTL23
NDUFB2
ESD
MYL6B
MRPL40
ATP5F1D
POLR2G
MTX1
LSM1
COMMD6
CMC2
IFITM2
GSTM3
OSTF1
MRPL22
MRPL23
GABARAPL2
IFITM1
UQCRHL
ATOX1
NUP205
DBNDD2
MSRB2
APOO
ANXA1
TMEM45A
HMGCL
PSMD9
REXO2
NUDT2
LOXL1
C3orf14
POP5
CDR1
NDUFA6
AATF
PSMB3
NDUFB1
DYNLL1
EIF3F
DNPH1
SLC25A4
GSTP1
TGIF2−RAB5IF
TIMM13
POLR2L
MT1X
MGST3
S100A11
PSMB5
TRMT112
PSMA6
HEXB
TIMP1
FAU
PSMB1
SSR4
NDUFB11
RPL36AL
ATP5MF
SNRPD3
MICOS10
RTRAF
MRPL51
ATP5MC2
ATP5MC1
MAP2K3
A84T
A84T
A84T
A84T
A84T - Tet
WT - Tet
A84T - Tet
WT
A84T - Tet
WT - Tet
WT
WT
WT
WT - Tet
WT - Tet
A84T - Tet
B
Enriched terms
mitochondrial protein-containing complex
mitochondrial inner membrane
organelle inner membrane
mitochondrion
mitochondrial envelope
mitochondrial membrane
envelope
organelle envelope
cytoplasm
inner mitochondrial membrane protein complex
nucleosome
DNA packaging complex
nucleus
protein-DNA complex
chromatin
intracellular membrane-bounded organelle
CENP-A containing nucleosome
CENP-A containing chromatin
chromosome, centromeric core domain
nucleoplasm
10
10
30
50
70
90
negative log10 (adj. p-value)
Gene expression change

Figure S8 - Additional gene expression contrasts

Gene expression volcano plots showing different control contrasts. All are between pairs of induced (+tet) or not-induced (-tet) TRex HEK293 cells transfected with particular constructs. A) MAP2K3 variants p.Thr222Met or p.Arg96Trp (each +tet) vs the appropriate -tet control or to MAP2K3 WT +tet. B) CHEK2 p.Thr68Ala +tet vs -tet or to CHEK2 WT +tet. C) MAP2K1 variants p.Gln56Pro, p.Lys97Ala or p.Val211Asp (+tet) vs the appropriate -tet control or to MAP2K1 WT +tet.

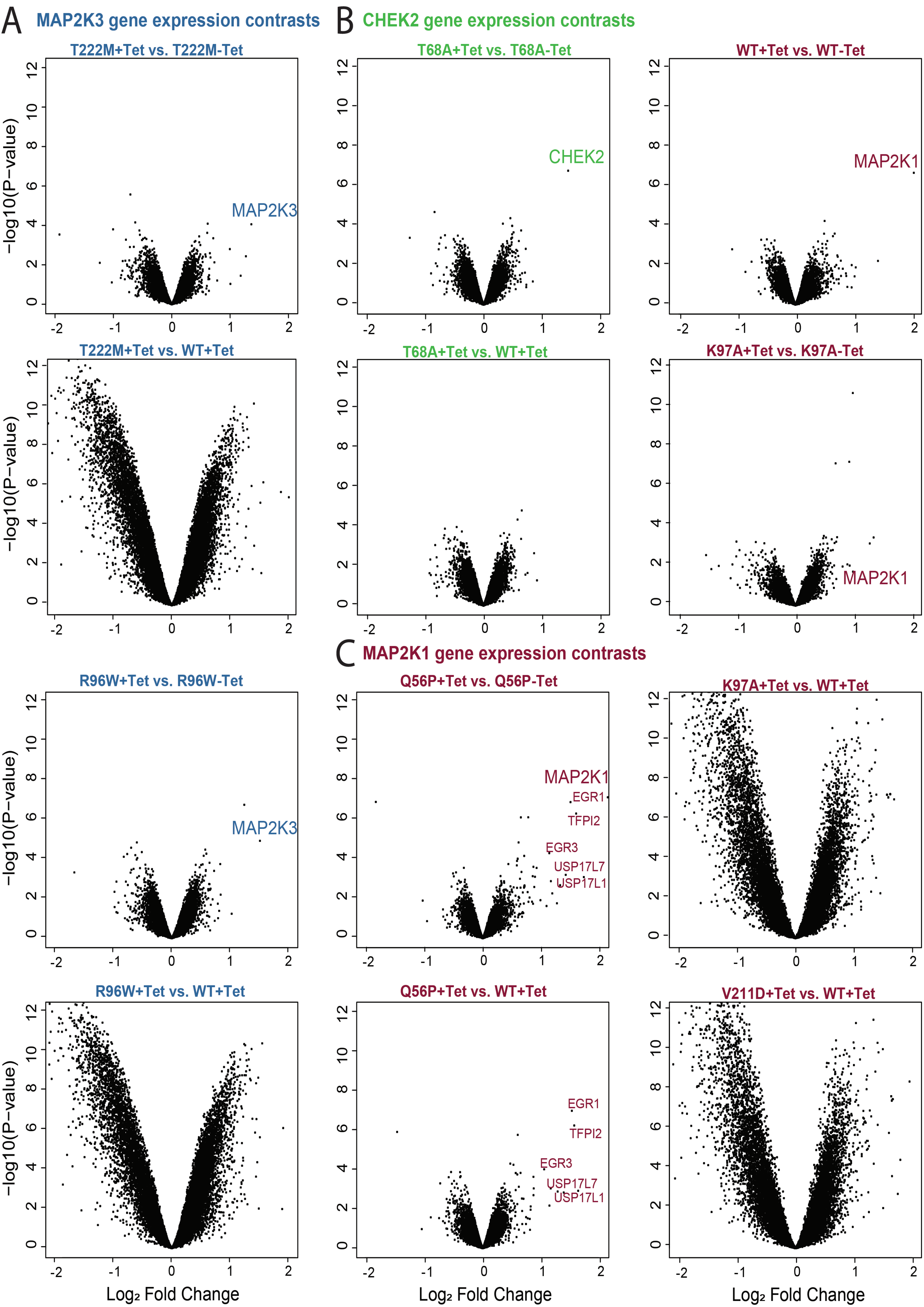
A MAP2K3 gene expression contrasts
B CHEK2 gene expression contrasts
C MAP2K1 gene expression contrasts
T222M+Tet vs. T222M-Tet
T222M+Tet vs. WT+Tet
R96W+Tet vs. R96W-Tet
R96W+Tet vs. WT+Tet
T68A+Tet vs. T68A-Tet
T68A+Tet vs. WT+Tet
WT+Tet vs. WT-Tet
K97A+Tet vs. K97A-Tet
Q56P+Tet vs. Q56P-Tet
Q56P+Tet vs. WT+Tet
K97A+Tet vs. WT+Tet
V211D+Tet vs. WT+Tet
MAP2K3
CHEK2
MAP2K1
EGR1
TFPI2
EGR3
USP17L7
USP17L1
−log10(P−value)
Log2 Fold Change

Figure S9 - MAP2K3 Mitotracker Staining

A) T-REx-293 cells transfected with MAP2K3 WT or p.Ala84Thr in presence (+tet) or absence (-tet) of tetracycline induction. Top row: Brightfield with 40x magnification taken on a Ti2 Nikon microscope and captured with a Nikon DS-Qi2 camera. Center row: Cells stained for 15 min with 100 nM Mitotracker Red CMXRos. Bottom row: Composite. B) Analysis of mitotracker signal. Mean brightness (grey value) of 50 - 80 cells per condition was measured using ImageJ 1.52p. Asterisk indicates $p \leq 0.05$.

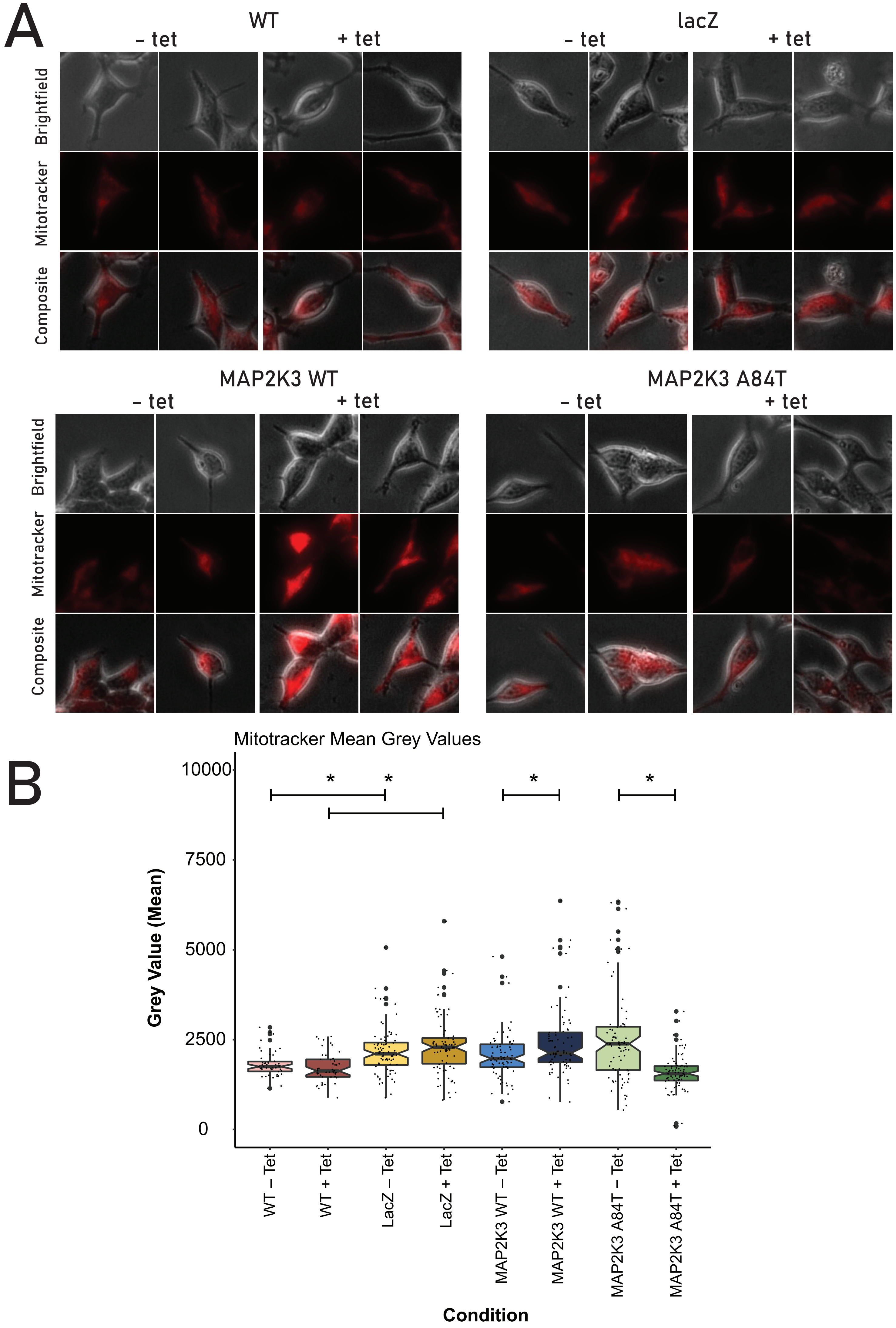
A
WT
– tet
+ tet
lacZ
– tet
+ tet
Brightfield
Mitotracker
Composite
MAP2K3 WT
– tet
+ tet
MAP2K3 A84T
– tet
+ tet
Brightfield
Mitotracker
Composite
B
Mitotracker Mean Grey Values
Grey Value (Mean)
10000
7500
5000
2500
0
*
*
*
*
WT – Tet
WT + Tet
LacZ – Tet
LacZ + Tet
MAP2K3 WT – Tet
MAP2K3 WT + Tet
MAP2K3 A84T – Tet
MAP2K3 A84T + Tet
Condition

Figure S10 - Additional gene expression controls

Left: Gene expression contrast of T-REx-293 +tet vs. -tet. Centre: T-REx-293 transfected with a lacZ control vector (+/-tet). Right: T-REx-293 transfected with a lacZ control vector + tet vs. T-REx-293 WT cells + tet.

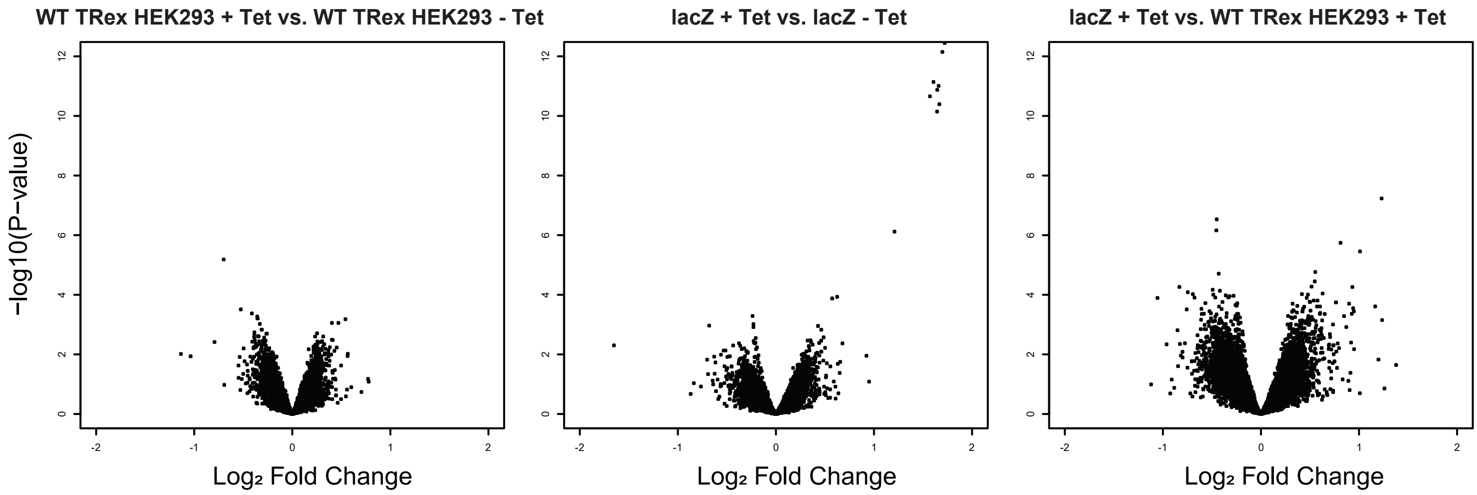
WT TRex HEK293 + Tet vs. WT TRex HEK293 - Tet
lacZ + Tet vs. lacZ - Tet
lacZ + Tet vs. WT TRex HEK293 + Tet
-log10(P−value)
0
2
4
6
8
10
12
-2
-1
0
1
2
Log₂ Fold Change
